# Continuous renal replacement therapy with the adsorptive oXiris filter may be associated with the lower 28-day mortality in sepsis: a systematic review and meta-analysis

**DOI:** 10.1186/s13054-023-04555-x

**Published:** 2023-07-09

**Authors:** Guizhong Wang, Yuxuan He, Qingling Guo, Ying Zhao, Jie He, Yue Chen, Weijia Chen, Yi Zhou, Zichong Peng, Ke Deng, Jianbin Guan, Wenting Xie, Ping Chang, Zhanguo Liu

**Affiliations:** Department of Critical Care Medicine, Zhujiang Hospital, The Second School of Clinical Medicine, Southern Medical University, 253 Gongye Rd, Guangzhou, 510282 China

**Keywords:** oXiris, Sepsis, Mortality, Systematic review, Meta-analysis

## Abstract

**Background:**

The oXiris is a novel filter for continuous renal replacement therapy (CRRT) featuring an adsorption coating to adsorb endotoxins and remove inflammatory mediators. Given that no consensus has been reached on its potential benefits in treating sepsis, a meta-analysis was conducted to assess its impact on the clinical outcomes of this patient population.

**Methods:**

Eleven databases were retrieved to find relevant observational studies and randomized controlled trials. The Newcastle–Ottawa Scale and the Cochrane Risk of Bias Tool were used to assess the quality of the included studies. The Grading of Recommendations, Assessment, Development, and Evaluation (GRADE) process was employed to assess the certainty of evidence. The 28-day mortality was the primary outcome. Secondary outcomes were 7-, 14-, and 90-day mortality, length of intensive care unit (ICU) and hospital stay, ICU and hospital mortality, norepinephrine (NE) dose, interleukin-6 (IL-6) and lactate levels, and Sequential Organ Failure Assessment (SOFA) score.

**Results:**

The meta-analysis, pooling data from 14 studies, involving 695 patients, showed significant reductions in 28-day mortality [odds ratio (OR) 0.53; 95% confidence interval (CI) 0.36–0.77, *p* = 0.001] and length of ICU stay [weighted mean difference (WMD) − 1.91; 95% CI − 2.56 to − 1.26, *p* < 0.001)] in patients with sepsis using the oXiris filter compared to other filters. Besides, the SOFA score, NE dose, IL-6 and lactate levels, and 7- and 14-day mortalities were lower in the oXiris group. However, the 90-day mortality, ICU and hospital mortality, and length of hospital stay were comparable. The quality assessment of the ten observational studies indicated intermediate to high quality (average Newcastle–Ottawa score: 7.8). However, all four randomized controlled trials (RCTs) had an unclear risk of bias. The evidence for all outcomes had a low or very low level of certainty because the original study design was mainly observational studies and the RCTs included had an unclear risk of bias and a small sample size.

**Conclusion:**

The treatment with the oXiris filter during CRRT in sepsis patients may be associated with lower 28-, 7-, and 14-day mortalities, lactate levels, SOFA score, NE dose, and shorter length of ICU stay. However, due to the low or very low quality of evidence, the effectiveness of oXiris filters was still uncertain. Besides, no significant difference was observed for the 90-day mortality, ICU and hospital mortality, and length of hospital stay.

**Supplementary Information:**

The online version contains supplementary material available at 10.1186/s13054-023-04555-x.

## Introduction

Sepsis is a severe condition characterized by uncontrolled host response to infection, leading to organ dysfunction and high mortality rates [1]. Its underlying pathogenesis is unclear, but studies have shown that bacterial endotoxins could trigger dysregulation and excessive cascade release of proinflammation, thereby inducing sepsis [2]. Therefore, strategies aimed at the efficient removal of endotoxins and inflammatory factors are considered an essential aspect of the comprehensive approach to treating sepsis and optimizing patient outcomes [3].

In recent years, continuous blood purification therapy has emerged as a treatment approach for sepsis. This therapy can potentially remove inflammatory cytokines from the bloodstream, thereby reducing the overall inflammatory response and promoting the restoration of immune homeostasis [4, 5]. The oXiris hemofilter, designed for endotoxin adsorption, incorporates a novel approach by utilizing the inherent hydrogel structure of the AN69 membrane. Additionally, the surface of the oXiris filter is coated with polyethyleneimine (PEI) and heparin coatings [6, 7]. PEI is a multilayer linear structure cationic complex that can adsorb endotoxins with negative charges on the surface. At the same time, the negative charges retained in the bottom layer can absorb inflammatory cytokines [6, 7]. It has been reported that the application of PEI on surfaces can enhance the endotoxin adsorption and cytokines removal capacity of the AN69 membrane [5]. Compared to other hemofilters, the oXiris hemofilter effectively liminates inflammatory mediators and adsorbs endotoxins.

Nonetheless, there is still debate on whether it brings advantages in treating patients with sepsis [8, 9]. Therefore, all published and ongoing studies were pooled together to assess the impact of the oXiris filter on clinical outcomes of this patient population.

## Methods

### Protocol and registration

The methods and reports of this study followed the Preferred Reporting Items for Systematic Reviews and Meta-Analyses guidelines (PRISMA 2020 Checklist) (Additional file 1). The study protocol was registered in the international PROSPERO database for systematic reviews (registration number CRD42022356340). Amendments to the information provided in the protocol are shown in Additional file 2: Appendix 9.

### Study inclusion and exclusion criteria

The criteria for the inclusion and exclusion of articles were as follows. The target population included adults diagnosed with sepsis undergoing CRRT. The intervention group included patients who received treatment with the oXiris filter, while the comparison group comprised patients who received other types of filter treatments. It is important to note that the oXiris group and the other filters group consisted of different individuals. Only observational studies (cohort studies and case series studies with controls) and randomized controlled trials (RCTs) were included. If studies lacked available data, they were excluded. If studies did not have a control group and used self-control before and after the intervention, they were also excluded.

### Literature research and data extraction

Eleven databases (English databases: Web of Science, the Cochrane Library, PubMed, and EMBASE; Chinese databases: Wanfang Database, China National Knowledge Infrastructure, Sino-Med, and VIP Database for Chinese Technical Periodicals; and three major clinical research registration websites: International Clinical Trials Registry Platform, Chinese Clinical Trial Registry, and ClinicalTrials.gov) were systematically searched to collect all studies relevant to this research as of July 2022. To account for the possibility of new literature being published during the meta-analysis and article writing process, an additional search was conducted across 11 databases from July 2022 to November 9, 2022. The detailed search strategy is shown in Additional file 2: Appendix 1.

### Study selection

The literature screening process involved three fellows who independently assessed the titles, abstracts, and full texts to determine whether the literature met the eligibility criteria. In cases of disagreements between the investigators, consensus-based decision-making was utilized. If necessary, a fourth investigator was consulted to resolve any disputes.

### Data extraction

Four researchers independently retrieved data from the included study and used standardized forms for consistency. The data extracted included study characteristics, patient characteristics, intervention measures, and results. The method for unknown non-normal distributions was applied to transform the median and quartile into the mean and standard deviation (SD) [10, 11]. Graphical data in the original studies were extracted using WebPlotDigitizer, a semiautomated tool that extracts underlying digital data by reverse engineering a visual image of the data [12, 13]. Disagreements during the data extraction process were settled by consensus among the four researchers.

### Outcomes

The primary outcome was the 28-day mortality, while the secondary outcomes included 7-, 14-, and 90-day mortality, length of ICU and hospital stay, ICU and hospital mortality, norepinephrine (NE) dose, lactate and interleukin-6 (IL-6) and lactate levels, and Sequential Organ Failure Assessment (SOFA) score.

### Assessment of risk of bias

Three investigators, respectively, evaluated the quality of the included studies. The Cochrane Risk of Bias Tool was used to assess the quality of the included RCTs [14]. Each area was given a risk of bias rating of low, unclear, or high. If a study had a high risk of bias for one or more key areas, it was considered at high risk of bias. If a study had an unclear risk of bias for one or more key areas and no high-risk areas, the study's risk of bias was considered unclear. If a study had a low risk of bias for all key areas, the risk of bias was considered low [15]. The Newcastle–Ottawa Scale (NOS) was used to assess the quality of the included observational studies [16]. Scores < 6 were classified as low quality, 6 to 7 as intermediate quality, and 8 to 9 as high quality. To evaluate the impact of a low-quality study on the results of the meta-analysis and whether the results were robust, it was included in meta-analyses but excluded for sensitivity analysis when conducting meta-analyses.

### Statistical analysis

Interventions in each group, outcomes, and sample sizes of each study were summarized in a table. Revman version 5.3 was used for the statistical analysis. The primary outcome was 28-day mortality.

The Mantel–Haenszel method was used to analyze the binary variable and was represented as odds ratio (OR). The weighted mean difference (WMD) was used as the effect indicator while analyzing the continuous variables using the inverse variance approach. The standardized mean difference (SMD) was utilized as the effect indicator when the absolute values of the continuous variables differed greatly, or the units were not uniform. All results were visualized in forest plots. Statistical significance was defined as a two-tailed *p* < 0.05. The statistical heterogeneity in the included trials was evaluated using I^2^ statistics and Cochrane's Q test. Heterogeneity was deemed significant when *I*^2^ statistics were greater than 50%, and the P value for Cochrane's Q test was less than 0.1. The fixed-effects model was applied for meta-analyses if I^2^ was less than or equal to 50%. The random-effects model was used for meta-analyses if I^2^ was more than 50%. A publication bias test was not conducted for outcomes that included < 10 articles to avoid misjudging accidental errors as publication bias due to the limited number of studies.

In Xie et al.'s study, the investigators employed the inverse probability of treatment-weighting method (IPTW) to reduce selection bias and control for confounding factors. After IPTW, the total sample size increased from 76 to 149 [17], which might inflate the weight of Xie's study in the entire meta-analysis, thus affecting the pooled results. Therefore, for 7-, 14-, and 28-day mortality, length of ICU and hospital stay, the sensitivity analysis method was used to separately include data before and after IPTW for two independent meta-analyses of each outcome. The results of the two meta-analyses were compared to judge whether the IPTW in Xie et al.'s study affected the meta-analysis results.

If the treatment period was inconsistent, the results after the longest treatment period of CRRT were included in the meta-analysis. For SOFA score, NE dose, lactate level, and IL-6 level, post-treatment data were included for meta-analyses, because the accurate mean and SD of data on changes before and after treatment could not be obtained. To explore the baseline similarity, meta-analyses on the baseline data of these four outcomes were conducted. If an included original study did not report the baseline data, its post-treatment data were excluded for sensitivity analysis to assess whether unknown baseline incomparable risk contributed to the results of the meta-analysis. If the results of the outcomes of the oXiris and control groups showed significant differences at baseline or data reported in a study could not be accurately converted into the mean and SD of the outcomes after treatment, the outcomes of the studies were not included for meta-analysis, but qualitatively described and compared to the meta-analysis of other studies.

For IL-6 level, NE dose, and SOFA score, only the post-IPTW data were used for meta-analysis when Xie et al.'s study provided data before and after IPTW [17].

### Assessment of the quality of evidence

The Grading of Recommendations, Assessment, Development, and Evaluation (GRADE) method was used to rate the quality of the evidence using the GRADEpro GDT online tool [18, 19]. The rating process referred to the GRADE Handbook and guidelines [20, 21]. When studies investigating outcomes included both RCTs and observational studies, subgroup analyses were conducted according to the type of studies. The quality of the evidence from RCTs and observational studies was rated using the GRADE method.

## Results

### Literature search

**Fig. 1:**
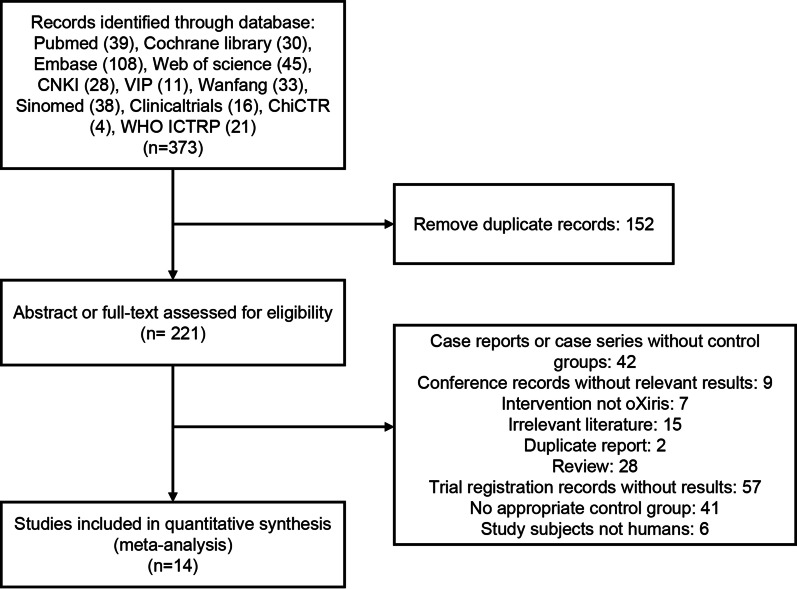
Flow diagram of study selection

The initial and supplemental search identified 373 publications. After removing 152 duplicate publications, 221 results were rescreened based on abstracts or full text. The detailed selection process is shown in Fig. 1. Finally, the quantitative meta-analysis included 14 publications that met the eligibility criteria [6, 17, 22–33].

### Description of the included studies

After literature screening, our meta-analysis included 14 publications that met the eligibility requirements, comprising 4 RCTs and 10 cohort studies. The controls in our meta-analysis were defined as filters other than oXiris. The 14 articles were published between 2013 and 2022, including 695 participants, with 304 patients in the oXiris group, and 391 in the control group. The filters used in each of the included studies are listed in Table 1. In addition, the characteristics of these filters are summarized in Additional File 2: Appendix 2. Furthermore, the age of these patients ranged from 55 to 65 years old, and the treatment duration varied across the articles. Thirteen of the studies were conducted in China and one from Sweden. Information on the demographics and features of the included studies are provided in detail in Table 1.

**Table 1:** Characteristics of included studies in the meta-analysis

Study	Year	Region	Study type	Filter	Age	M	F	Infection Site	APACHE II Score	SOFA Score	Treatment Duration (hour)	Efficacy
Zang [22]	2020	China	CS	oXiris	59.5 [31, 80]	5	5	Ia (7/10); P&O (3/10)	21 ± 8.2	11.7 ± 4.2	39.5 [27, 74]	⑨⑫
				AN69-ST	58 [29, 78]	5	7	Ia (9/12); P&O (3/12)	19.7 ± 5.9	11.8 ± 4.5	42 [25, 86]	
Shum [23]	2013	China	CS	oXiris	62 (58, 73)	4	2	Ia (5/6); O (1/6)	36 (28, 41)	12 (9, 15)	20.7 (10.3, 36.2)	⑤⑥⑦⑧⑫
				FX80	76 (66, 81)	15	9	Ia (23/24); O (1/24)	34 (31, 37)	13 (10, 15)	20.4 (8.7, 34.7)	
Zang [24]	2022	China	CS	oXiris	55 (45, 73.5)	12	10	Ia (16/22); P (3/22); O (3/22)	16.50 (14.50, 25.25)	11.00 (9.00, 12.25)	48 (39, 66.25)	⑤⑦⑧⑨⑪⑫
				AN69-ST	57 (44.5, 69.5)	11	11	Ia (14/22); P (3/22); O (5/22)	17.00 (12.00, 22.00)	11.00 (9.00, 13.50)	48 (38.5, 70.5)	
Guan [25]*	2022	China	CS	oXiris	56.37 ± 15.06	48	22	Ia (26/70); P (58/70); S/T (9/70); U (8/70); Bl (32/70); CVM (4/70); O (3/70)	–	16.97 ± 3.56	Not mentioned	①②③④⑤⑦⑪⑫
				AN69-ST150	54.42 ± 12.75	50	16	Ia (30/66); P (57/66); S/T (7/66); U (11/66); Bl (10/66); O (5/66)	–	14.98 ± 3.15		
Feng [26]	2022	China	RCT	oXiris	62.4 ± 17.4	5	3	Not mentioned	19.9 ± 6.6	9.9 ± 2.9	20.3 (11.3, 50.6)	③⑤⑦⑪
				AN69-ST	69.1 ± 12.6	6	2		23.1 ± 4.2	9 ± 1.5	24.5 (14.5, 46.8)	
Xie [17]	2022	China	CS	oXiris	64.83 ± 17.64	21	9	Not mentioned	21.80 ± 7.13	–	Not mentioned	①②③⑤⑦⑨
				M100	67.07 ± 16.95	34	12		26.98 ± 6.99	–		
Broman [6]	2019	Sweden	RCT	oXiris	[53, 82]	7	1	Ia (7/8); U (1/8)	–	–	24	⑨⑩⑪⑫
				AN69-ST	[54, 80]	3	5	Ia (3/8); O (5/8)	–	–	24	
Zhai [27]	2021	China	CS	oXiris	59.73 ± 13.02	13	10	Not mentioned	21.46 ± 2.55	12.64 ± 2.85	8–10	⑤⑨⑩⑪⑫
				conventional filter	58.97 ± 12.51	18	12		21.58 ± 3.10	12.97 ± 3.01		
Le [28]	2022	China	CS	oXiris	55.7 ± 15.0	13	5	Not mentioned	19.2 ± 4.1	8.6 ± 1.7	Not mentioned	①③④⑤
				AN69-ST100	58.1 ± 14.3	27	14		17.6 ± 5.1	7.9 ± 2.2		
He [29]	2019	China	RCT	oXiris	48.67 ± 12.87	24	6	Not mentioned	23.567 ± 5.51	–	Not mentioned	③⑤⑧⑨⑪
				AN69-ST	49.27 ± 11.68	22	8		23.533 ± 2.91	–		
Yu [30]	2020	China	CS	oXiris	57.6 ± 13.4	12	8	Ia (9/20); U (7/20); Bi (4/20)	18.9 ± 2.7	12.8 ± 1.8	4.8 ± 1.8	⑤⑥⑦⑧⑨⑩⑪⑫
				AN69-ST	52.5 ± 14.8	14	11	Ia (13/25); U (8/25); Bi (4/25)	18.1 ± 2.6	12.3 ± 2.3	6.6 ± 2.1	
Lin [31]*	2021	China	CS	oXiris	64.83 ± 17.64	11	4	Ia (4/15); P (14/15); S/T (2/15); U (1/15); O (1/15)	19.5 ± 4.5	11.8 ± 5.6	Not mentioned	①②③⑨⑪⑫
				M150	67.07 ± 16.95	19	11	Ia (15/30); P (19/30); S/T (3/30); U (5/30); O (2/30)	18.9 ± 6.3	10.7 ± 5.8		
Kang [32]	2022	China	CS	oXiris	59.50 (45.00, 68.25)	11	3	Ia (2/14); P (8/14); O (4/15)	18.92 ± 2.400	18.00 ± 2.253	48	③⑧⑨⑩⑪⑫
				conventional filter	59.50 (55.00, 69.00)	9	10	Ia (12/19); P (3/19); O (5/19)	19.57 ± 4.537	17.15 ± 3.848	48	
Lu [33]	2022	China	RCT	oXiris	43.2 ± 24.1	15	15	Not mentioned	–	12.60 ± 3.30	48	⑨⑩⑫
				AN69-ST	43.1 ± 24.2	13	17		–	12.50 ± 3.70	48	

*CS*: Cohort study; *M* Sample size of males; *F* Sample size of females; *RCT*: Randomized controlled trial; *Ia*: Intra-abdominal; *P*: Pulmonary; *U*: Urinary; *Bi*: Biliary; *S/T*: Skin or tissue; *Bl*: Blood; *CVM*: Cardiac valves myocardium; *O*: Other; *LOS*: Length of stay

①7-day mortality, ②14-day mortality, ③28-day mortality, ④90-day mortality, ⑤ICU LOS, ⑥ICU mortality, ⑦Hospital LOS, ⑧Hospital mortality, ⑨lactic acid, ⑩noradrenaline dose, ⑪interleukin-6, ⑫Sequential Organ Failure Assessment (SOFA) Score

The Data are shown as *Mean* ± *SD*, *Median (Interquartile Range) or Median [Min, Max]*

*The presence of more than one infection site in some patients is mentioned in these articles

### Risk of bias

**Fig. 2:**
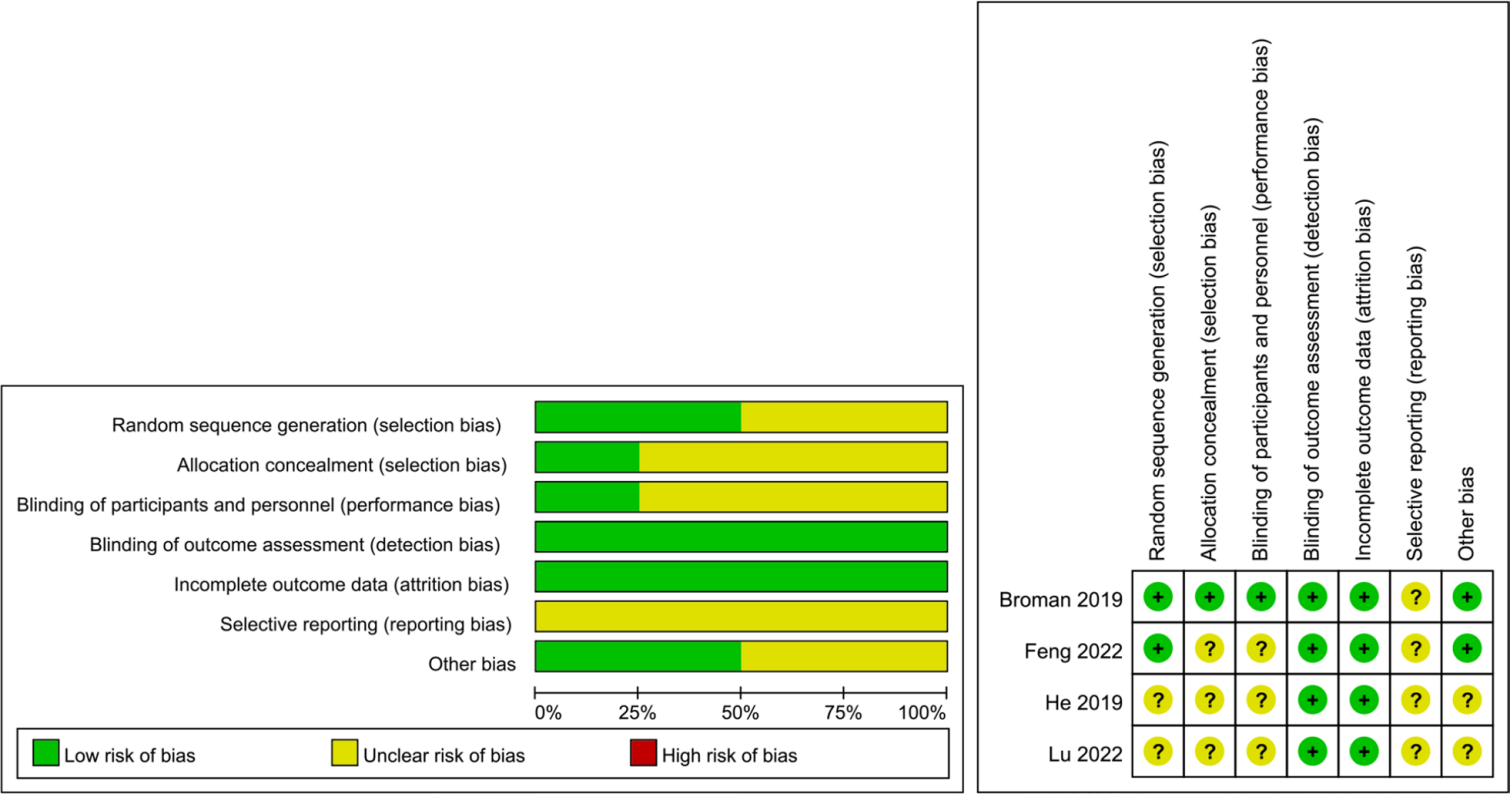
Risk of bias summary

The average Newcastle-Ottawa score of all ten observational studies was 7.8 ± 0.8 (Mean ± SD) indicating intermediate- or high-quality studies (Additional file 2: Appendix 3) [17, 22–25, 27, 28, 30–32]. However, when the Cochrane Risk of Bias Tool was used to assess four RCTs, all of them were rated as having an unclear risk of bias due to unclear risk of bias for one or more key areas and no high-risk areas (Fig. 2). Notably, studies by He et al. and Lu et al. contributed to a significant amount of unclear information regarding bias assessment [29, 33].

### Primary outcome

**Fig. 3.**
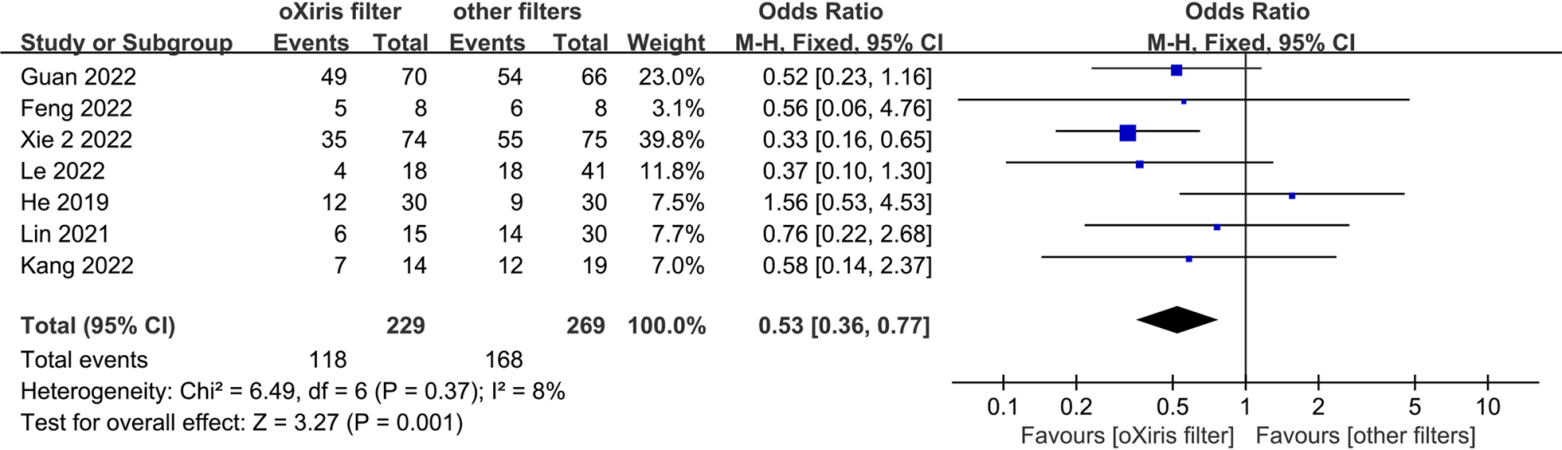
28-day mortality (adopting Xie et al.'s data after IPTW)

The pooled meta-analysis results showed that the oXiris filter was associated with significantly lower 28-day mortality in sepsis patients than other filters [adopting Xie et al.'s data after IPTW: OR 0.53; 95% CI 0.36–0.77, *p* = 0.001, *I*^2^ = 8%; Fig. 3]. The findings of the heterogeneity test indicated that studies with 28-day mortality had acceptable heterogeneity (I^2^ < 50%). This result was consistent with sensitivity analyses adopting Xie et al.'s data before IPTW (Additional file 2: Fig. S3, Appendix 4).

### Secondary outcomes

The meta-analysis demonstrated that the oXiris filter was associated with lower 7- and 14-day mortalities than other filters (Additional file 2: Figs. S1, S2, S6, S7, Appendices 4, 5).

**Fig. 4:**
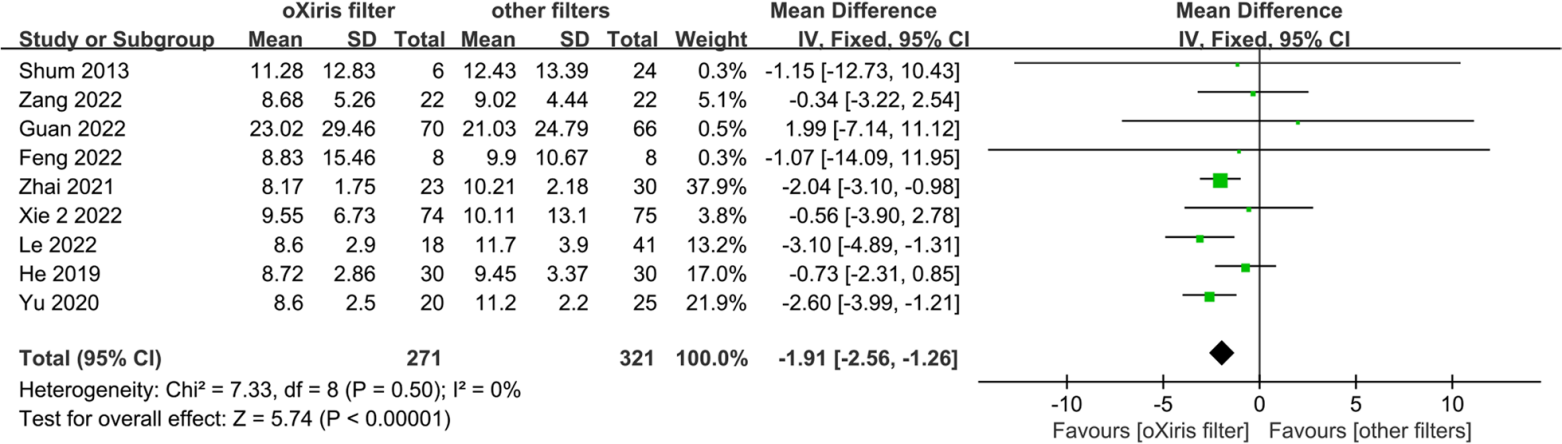
The length of ICU stay (adopting Xie et al.'s data after IPTW)

The length of ICU stay (adopting Xie's data after IPTW: WMD − 1.91; 95% CI − 2.56 to − 1.26, *p* < 0.001, *I*^2^ = 0%; Fig. 4) was shorter in the oXiris group compared to the other filters control group. No significant heterogeneity was found among the studies. This result was consistent with sensitivity analyses adopting Xie et al.'s data before IPTW (Additional file 2: Fig. S4, Appendix 4).

The 90-day mortality, ICU mortality, hospital mortality, and the length of hospital stay were comparable between the oXiris group and the control group (Additional file 2: Figs. S5, S8–S11, Appendices 4, 5).

Four meta-analyses were conducted on the baseline data of SOFA score, NE dose, lactate level, and IL-6 level to explore the baseline similarity. In each of the original studies included in the four meta-analyses, there was no significant difference in these four outcomes between the oXiris group and the control group. The results of these meta-analyses also showed that they were comparable between the two groups at baseline (Additional file 2: Figs. S27–S30, Appendix 10).

**Fig. 5:**
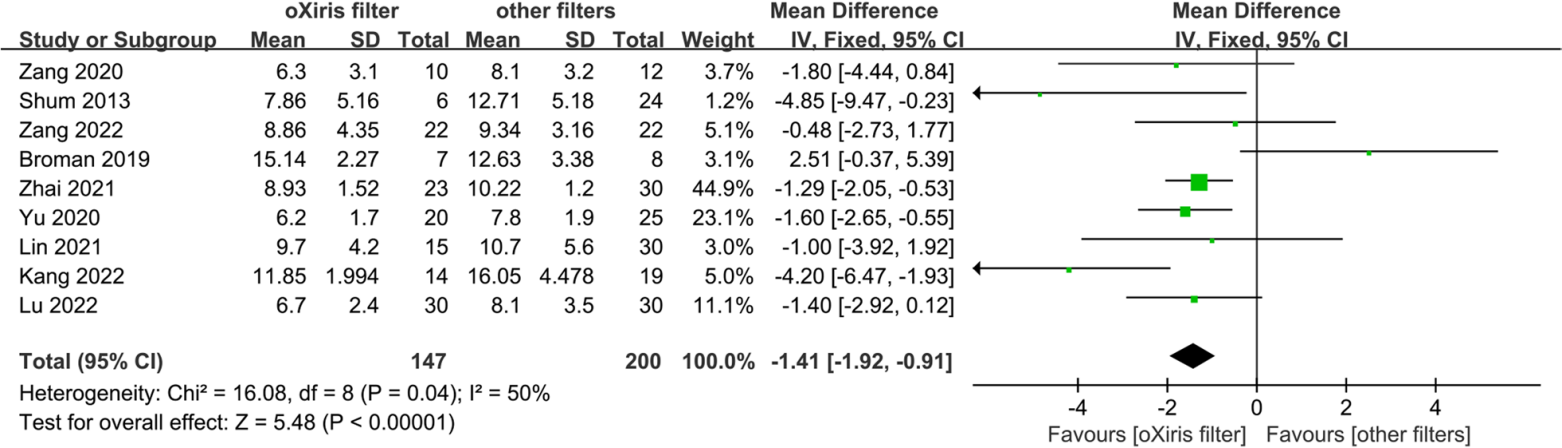
SOFA score

For the SOFA score, the meta-analysis of nine trials involving 347 patients found that oXiris filters were associated with significantly lower SOFA scores compared to other filters (WMD − 1.41; 95% CI − 1.92 to − 0.91, *p* < 0.001, *I*^2^ = 50%; Fig. 5) [6, 22–24, 27, 30–33]. This result was consistent with sensitivity analysis excluding Broman et al.’s study that did not report baseline SOFA score (Additional file 2: Fig. S31, Appendix 10) [6]. Data from Guan et al.'s study were not included in the meta-analysis due to significant differences at baseline [25]. Guan et al.'s study found that a 48-h treatment of CRRT with the oXiris filter was associated with a significantly lower SOFA score than the AN69-ST150 filter [25].

**Fig. 6:**
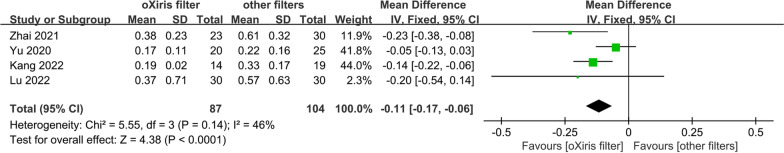
NE dose

**Fig. 7:**
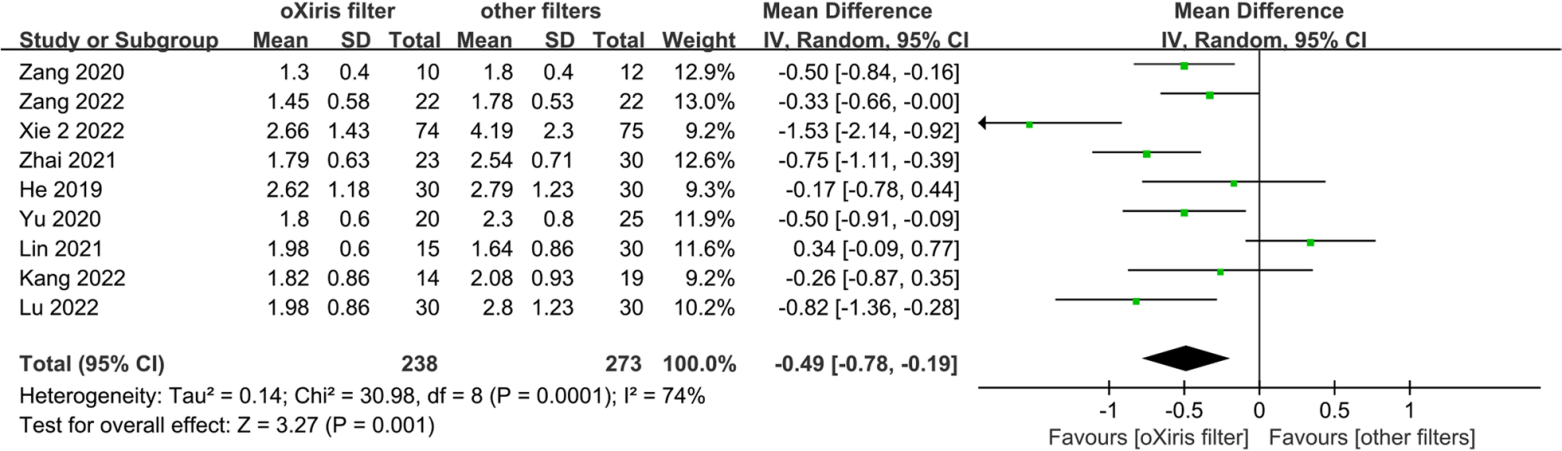
Lactate level

Regarding hemodynamic variables, the pooled analysis of four studies of 191 patients indicated that the use of oXiris filters was associated with a significantly lower dosage of NE (μg/(kg·min)) compared to other filters (WMD -0.11; (95% CI − 0.17, to − 0.06; *p* < 0.001, *I*^2^ = 46%; Fig. 6) [27, 30, 32, 33]. Besides, the meta-analysis of nine trials involving 511 patients showed that the oXiris filter significantly decreased the lactate level (mmol/L) in patients with sepsis compared to other filters (WMD =  − 0.49; 95% CI =  − 0.78, to − 0.19, *p* = 0.001, *I*^2^ = 74%; Fig. 7) [17, 22, 24, 27, 29–33]. This result was consistent with sensitivity analysis excluding Xie et al.’s study that did not report baseline lactate level (Additional file 2: Fig. S32, Appendix 10) [17]. Data from Broman et al.'s study were not included in these two meta-analyses due to the unavailability of the accurate mean and SD of the outcomes after treatment. In Broman et al.’s study, it was found that a 24-h treatment of CRRT using the oXiris filter was associated with significantly lower lactate levels (*P* = 0.02) and NE dose (*P* = 0.02) compared to the standard filter [6].

**Fig. 8:**
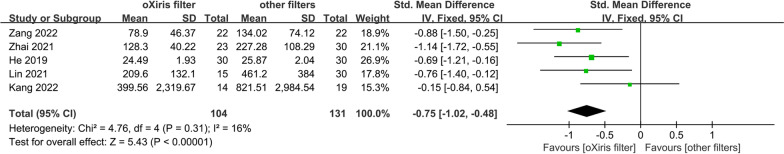
IL-6 level

Five studies involving 235 patients reported data on IL-6 levels. The pooled estimates revealed that oXiris filters were associated with significantly lower IL-6 levels (pg/ml) compared to other filters (SMD − 0.75; 95% CI − 1.02 to − 0.48, *p* < 0.001, *I*^2^ = 16%; Fig. 8) [24, 27, 29, 31, 32]. The data of another four studies were not included in the meta-analysis due to significant differences at baseline or the unavailability of the accurate mean and SD of the outcomes after treatment [6, 25, 26, 30]. In three of them, the oXiris group was associated with significantly lower IL-6 levels than the control group [6, 26, 30]. In Guan et al.'s study, after a 24-h treatment period, the oXiris group experienced a greater reduction in IL-6 level than the AN69-ST100 filter. However, there was no significant difference after the 48-h or 72-h treatment period [25].

The subgroup and sensitivity analysis results are detailed in Additional File 2: Appendices 6 and 7.

### Certainty of evidence

The evidence for all outcomes had a low or very low level of certainty because the original study design was mainly observational studies, and the RCTs included had an unclear risk of bias and a small sample size. Further, evidence from observational studies or RCTs was analyzed independently. Only the certainty of the evidence from observational studies for 7-, 14-, and 28-day mortalities and length of ICU stay was low after rating with the GRADE method. The certainty of the evidence for other outcomes was very low (Additional file 2: Appendix 8).

## Discussion

This meta-analysis included 695 patients with sepsis that underwent CRRT. Our results showed that compared to other filters (AN69-ST, M100, M150 and FX80), the use of the oXiris filter during CRRT treatment may be associated with lower 7-, 14- and 28-day mortalities, SOFA score, NE dose, lactate level, and shorter length of ICU stay. However, the evidence for these outcomes had a low or very low level of certainty because the original study design was mainly observational studies and the RCTs included had an unclear risk of bias and a small sample size.

In Xie et al.'s study, the investigators employed the IPTW method to reduce selection bias and control for confounding factors [17]. To assess the potential impact of the increased virtual sample size resulting from IPTW in Xie et al.’s study on the meta-analysis results, the data before and after IPTW were separately included in two independent meta-analyses for each of the five outcomes: 7-day mortality, 14-day mortality, 28-day mortality, length of hospital stay, and length of ICU stay. The pooled results showed no significant difference using data before or after IPTW.

In the present study, we conducted a subgroup analysis of outcomes using Xie et al.'s data after IPTW. The outcomes analyzed included 28-day and hospital mortality, SOFA score, lactate level, IL-6 level, NE dose, length of hospital stay, and length of ICU stay. The subgroup analysis was performed according to the study design (i.e., RCTs vs. observational studies) if the outcomes were present in both types of studies. The certainty of the evidence was rated with the GRADE method. According to the subgroup analysis based on observational studies, oXiris was associated with a shorter length of ICU stay and lower 28-day mortality than other filters in patients with sepsis. And the certainty of the evidence was low. However, the meta-analysis of RCTs showed that oXiris did not significantly differ from other filters regarding 28-day mortality or length of ICU stay (*P* > 0.05). The very low certainty of the evidence may be attributed to the risk of bias, the small sample size of the RCTs, and imprecision. Given that the certainty of evidence from observational studies was higher than that from RCTs, it was inferred that oXiris was associated with lower 28-day mortality and shorter length of ICU stay in patients with sepsis. The meta-analysis of observational studies indicated that the oXiris filter showed reductions in the SOFA score, lactate level, and NE dose compared to other filters. However, no significant differences were observed when considering the findings from the meta-analysis of RCTs. The lack of significant variance during the analysis of RCTs may be attributed to the small sample sizes of the included RCTs. Evidence from RCTs or observational studies had very low levels of certainty. Based on the available evidence, the pooled results of all observational studies and RCTs suggested potential reduction effects with oXiris on the SOFA score, lactate level, and NE dose compared to other filters. However, the very low certainty of the evidence from both types of studies suggests that more high-quality RCTs are needed to confirm these findings. The meta-analysis of either RCTs or observational studies suggests a potential reduction effect of oXiris compared to other filters on the IL-6 level. However, the certainty of the evidence from both types of studies was very low. It is important to note that Guan et al.'s study, excluded from the meta-analysis due to significant baseline differences, did not find a lower IL-6 level with CRRT using oXiris for 72 h. Therefore, the effectiveness of the oXiris filter in reducing the IL-6 level remains controversial.

In three studies, the interleukin-8 (IL-8) level in the oXiris group was lower than in the control group and showed significant differences [6, 24, 30]. Furthermore, the oXiris-CVVH group's vasoactive inotropic score (VIS) was significantly lower than the control group in three studies [17, 24, 25]. Two of the included studies showed that the lower serum endotoxin degree was significantly associated with the oXiris filter group after treatment compared to other filters in CRRT [6, 27].

Moreover, we found that the length of hospital stay, ICU and hospital mortality, and 90-day mortality did not differ significantly, possibly due to small sample sizes and insufficient statistical efficiency. The evidence for these outcomes had a low or very low level of certainty primarily due to the small sample size and unclear risk of bias of most observational studies and RCTs.

In vitro studies have shown that oXiris could remove both endotoxin and cytokines compared to CytoSorb and Toraymyxin [7]. The adsorption of endotoxins and cytokines by the oXiris membrane could reduce the inflammation of the organism and rapidly alleviate the cascade response caused by inflammatory factors, thus improving the patient's outcome [27]. Current adsorptive blood purification techniques have been observed to improve hemodynamics and the respiratory system [34–36]. They involve the removal of bacterial toxins and cytokines from the patient's blood, suppressing excessive inflammatory responses, and restoring immune homeostasis [4]. The cellular theory proposes that adsorptive blood purification techniques can interact with immune cells and the blood filter, leading to various cellular responses. These blood purification methods can potentially regulate the expression of molecules on the surface of leukocytes involved in adhesion and migration, antigen presentation, and apoptosis. Some immune cells, such as neutrophils and monocytes, can also adhere to blood purification tools, contributing to regulating the immune system [5]. The oXiris membrane and other adsorptive blood purification techniques hold significant implications for treating sepsis patients by modulating immune cell responses and promoting immune system balance.

Low or very low quality of evidence from the present study suggested that compared with other filters (AN69-ST, M100, M150 and FX80), the use of the oXiris filter may be associated with better hemodynamic improvements (NE dose, and lactate level), improvement in organ function (SOFA score), shorter length of ICU stay, and lower short-term mortality. However, the oXiris filter did not bring benefits in reducing long-term mortality (90-day mortality), which may be attributed due to the small sample size. Overall, the oXiris filter may be promising for CRRT in sepsis patients. However, due to the small sample size and limited quality of the original studies, the role of oXiris remains to be further explored through future high-quality studies.

### Strengths and limitations

The therapeutic impact of CRRT treatment with the oXiris filter in patients with sepsis was initially investigated in this meta-analysis providing the basis for the clinical use of oXiris in treating sepsis. However, the limitations of this study should be acknowledged. First, most included studies were observational studies, and only four were RCTs. However, RCTs were conducted at single centers with small sample sizes. Accordingly, there is an urgent need for high-quality RCTs with sizable sample sizes. Second, for SOFA score, NE dose, lactate level, and IL-6 level, the accurate mean and SD of data on changes in outcomes before and after treatment could not be obtained. Therefore, meta-analyses were not performed on the changes before and after treatment, but on baseline data and post-treatment data, respectively. Third, during the pooled analysis of IL-6 and lactate levels, NE dose, and SOFA score, some studies lacked accurate data conversion into the mean and SD of the outcomes after treatment, or had uneven baseline characteristics. As a result, it was not possible to pool data from all studies for a meta-analysis of these outcomes, and a qualitative description was provided instead. Fourth, there was high heterogeneity across studies on lactate level analysis (*I*^2^ > 50%). In addition, the regional limitation of the included studies should be acknowledged since most of them were from China. This limitation could not be addressed in the present study due to the lack of original studies. Nevertheless, there are ongoing RCTs in Switzerland and France (Registration nos.: NCT01948778 and NCT03426943) evaluating the efficacy of the oXiris filter in sepsis. With the publication of these studies and the subsequent updates of meta-analyses, the issue of regional bias may be addressed.

## Conclusion

Using the oXiris filter during CRRT treatment may be associated with lower 28-, 7-, and 14-day mortalities, SOFA score, NE dose, lactate levels, and shorter of ICU stay. However, due to the low or very low quality of evidence, the effectiveness of oXiris filters was still uncertain. Besides, it may not benefit in the 90-day mortality, ICU, hospital mortality, and length of hospital stay. High-quality RCTs with sizable sample sizes are warranted to validate the impact of the oXiris filter.

### Supplementary Information

**Additional file 1**: PRISMA 2020 checklist

**Additional file 2**: **Appendix 1**. The details of the search strategy; **Appendix 2**. Characteristics of different filters; **Appendix 3**. Risk of bias assessment; **Appendix 4**. Forest plots of outcomes adopting Xie’s data before IPTW; **Appendix 5**. Results of secondary outcomes; **Appendix 6**. Subgroup analyses of primary and secondary outcomes; **Appendix 7**. Sensitivity analyses by excluding low-quality studies; **Appendix 8**. Certainty of evidence; **Appendix 9**. Amendments to the information provided in the protocol; **Appendix 10**. The baseline similarity

## Data Availability

All data and materials related to our study are available by contacting the corresponding author.

## Abbreviations

CI: Confidence interval
CRRT: Continuous renal replacement therapy
GRADE: Grading of recommendations assessment, development, and evaluation
ICU: Intensive care unit
IL-6: Interleukin-6
IL-8: Interleukin-8
IPTW: Inverse probability weighting
NE: Norepinephrine
NOS: Newcastle–Ottawa Scale
OR: Odds ratio
PEI: Polyethyleneimine
CT: Randomized controlled trial
SMD: Standardized mean difference
SOFA: Sequential Organ Failure Assessment
VIS: Vasoactive-inotropic score
WMD: Weighted mean difference
