# Supplementary material for: Continuous renal replacement therapy with the adsorptive oXiris filter may be associated with the lower 28-day mortality in sepsis: a systematic review and meta-analysis
Source: Crit Care. 2023 Jul 9;27:275. doi: 10.1186/s13054-023-04555-x (PMC10331993; doi:10.1186/s13054-023-04555-x)
Supplement: Supplementary file 2 — Additional file 2: Appendix 1. The details of the search strategy; Appendix 2. Characteristics of different filters; Appendix 3. Risk of bias assessment; Appendix 4. Forest plots of outcomes adopting Xie’s data before IPTW; Appendix 5. Results of secondary outcomes; Appendix 6. Subgroup analyses of primary and secondary outcomes; Appendix 7. Sensitivity analyses by excluding low-quality studies; Appendix 8. Certainty of evidence; Appendix 9. Amendments to the information provided in the protocol; Appendix 10. The baseline similarity [file 13054_2023_4555_MOESM2_ESM.docx]

**Additional File 2**

[Appendix 1: The details of the search strategy 2](#_Toc139559784)

[Appendix 2: Characteristics of different filters 8](#_Toc139559785)

[Appendix 3: Risk of bias assessment 9](#_Toc139559786)

[Appendix 4: Forest plots of outcomes adopting Xie’s data before IPTW 10](#_Toc139559787)

[Appendix 5: Results of secondary outcomes 11](#_Toc139559788)

[Appendix 6: Subgroup analyses of primary and secondary outcomes 14](#_Toc139559789)

[Appendix 7: Sensitivity analyses by excluding low-quality studies 18](#_Toc139559790)

[Appendix 8: Certainty of evidence 20](#_Toc139559791)

[Appendix 9: Amendments to the information provided in the protocol 31](#_Toc139559792)

[Appendix 10: The baseline similarity 32](#_Toc139559793)

Appendix 1: The details of the search strategy

| **Search strategy** | **Result** |
| --- | --- |
| **Pubmed** |  |
| (Sepsis[MeSH Terms] OR Systemic Inflammatory Response Syndrome[MeSH Terms] OR Septic Shock[MeSH Terms] OR Multiple Organ Failure[MeSH Terms] OR Sepsis OR Systemic Inflammatory Response Syndrome OR SIRS OR Septic OR Toxic Shock OR Endotoxic Shock OR Endotoxin Shock OR Endotoxin Shocks OR (Inflammatory Response Syndrome, Systemic) OR Multiple Organ Dysfunction Syndrome OR Multiple Organ Failure OR Multiple Organ Failures OR MODS OR bloodstream infection OR bloodstream infections OR infection bloodstream OR Pyemia OR Pyemias OR Pyohemia OR Pyohemias OR Pyaemia OR Septicemia OR Septicemias OR (Poisoning, Blood) OR (Poisonings, Blood) OR poisoning blood OR blood poisoning OR blood poisonings OR severe sepsis OR sepsis severe OR Bacteremia OR Fungemia) AND oXiris  The first search was conducted from the establishment of the library to July 2022, and 36 articles were retrieved.  A supplementary search was conducted from July 2022 to November 9, 2022, and 3 articles were retrieved. | 39 |
| **The Cochrane Library** | |
| #1 MeSH descriptor: [Sepsis] explode all trees | 4959 |
| #2 MeSH descriptor: [Systemic Inflammatory Response Syndrome] explode all trees | 5354 |
| #3 MeSH descriptor: [Shock, Septic] explode all trees | 1082 |
| #4 MeSH descriptor: [Multiple Organ Failure] explode all trees | 442 |
| #5 Sepsis OR Systemic Inflammatory Response Syndrome OR SIRS OR Septic OR Toxic Shock OR Endotoxic Shock OR Endotoxin Shock OR Endotoxin Shocks OR Inflammatory Response Syndrome, Systemic OR Multiple Organ Dysfunction Syndrome OR Multiple Organ Failure OR Multiple Organ Failures OR MODS OR bloodstream infection OR bloodstream infections OR infection bloodstream OR Pyemia OR Pyemias OR Pyohemia OR Pyohemias OR Pyaemia OR Pyaemias OR Septicemia OR Septicemias OR Poisoning, Blood OR Poisonings, Blood OR poisoning blood OR blood poisoning OR blood poisonings OR severe sepsis OR sepsis severe OR Bacteremia OR Fungemia | 24277 |
| #6 Oxiris | 36 |
| #7 #1 or #2 or #3 or #4 or #5 | 25227 |
| #8 #6 and #7  The first search was conducted from the establishment of the library to July 2022, and 28 articles were retrieved.  A supplementary search was conducted from July 2022 to November 9, 2022, and 2 articles were retrieved. | 30 |
| **Embase** | |
| #1. 'sepsis'/exp OR 'systemic inflammatory response  syndrome'/exp OR 'septic shock'/exp OR 'multiple  organ failure'/exp | 366,821 |
| #2. 'sepsis'/exp OR sepsis OR 'systemic inflammatory response syndrome'/exp OR 'systemic inflammatory response syndrome' OR sirs OR septic OR 'toxic shock'/exp OR 'toxic shock' OR 'endotoxic shock'/exp OR 'endotoxic shock' OR 'endotoxin shock'/exp OR 'endotoxin shock' OR 'endotoxin shocks' OR 'inflammatory response syndrome, systemic' OR 'multiple organ dysfunction syndrome'/exp OR 'multiple organ dysfunction syndrome' OR 'multiple organ failure'/exp OR 'multiple organ failure' OR 'multiple organ failures' OR mods OR 'bloodstream infection'/exp OR 'bloodstream infection' OR 'bloodstream infections'/exp OR 'bloodstream infections' OR 'infection bloodstream' OR 'pyemia'/exp OR pyemia OR pyemias OR 'pyohemia'/exp OR pyohemia OR pyohemias OR 'pyaemia'/exp OR pyaemia OR 'septicemia'/exp OR septicemia OR septicemias OR 'poisoning, blood' OR 'poisonings, blood' OR 'poisoning blood' OR 'blood poisoning' OR 'blood poisonings' OR 'severe sepsis'/exp OR 'severe sepsis' OR 'sepsis severe' OR 'bacteremia'/exp OR bacteremia OR 'fungemia'/exp OR fungemia | 481,996 |
| #3. oxiris | 176 |
| #4. #1 OR #2 | 481,996 |
| #5. #3 AND #4  The first search was conducted from the establishment of the library to July 2022, and 102 articles were retrieved.  A supplementary search was conducted from July 2022 to November 9, 2022, and 6 articles were retrieved. | 108 |
| **Web of science** | |
| (Sepsis[MeSH Terms] OR Systemic Inflammatory Response Syndrome[MeSH Terms] OR Septic Shock[MeSH Terms] OR Multiple Organ Failure[MeSH Terms] OR Sepsis OR Systemic Inflammatory Response Syndrome OR SIRS OR Septic OR Toxic Shock OR Endotoxic Shock OR Endotoxin Shock OR Endotoxin Shocks OR (Inflammatory Response Syndrome, Systemic) OR Multiple Organ Dysfunction Syndrome OR Multiple Organ Failure OR Multiple Organ Failures OR MODS OR bloodstream infection OR bloodstream infections OR infection bloodstream OR Pyemia OR Pyemias OR Pyohemia OR Pyohemias OR Pyaemia OR Septicemia OR Septicemias OR (Poisoning, Blood) OR (Poisonings, Blood) OR poisoning blood OR blood poisoning OR blood poisonings OR severe sepsis OR sepsis severe OR Bacteremia OR Fungemia) AND oXiris  The first search was conducted from the establishment of the library to July 2022, and 43 articles were retrieved.  A supplementary search was conducted from July 2022 to November 9, 2022, and 2 articles were retrieved. | 45 |
| **Clinicaltrials** | |
| (Sepsis OR Systemic Inflammatory Response Syndrome OR Septic Shock OR Multiple Organ Failure OR Sepsis OR Systemic Inflammatory Response Syndrome OR SIRS OR Septic OR Toxic Shock OR Endotoxic Shock OR Endotoxin Shock OR Endotoxin Shocks OR Inflammatory Response Syndrome, Systemic OR Multiple Organ Dysfunction Syndrome OR Multiple Organ Failure OR Multiple Organ Failures OR MODS OR bloodstream infection OR bloodstream infections OR infection bloodstream OR Pyemia OR Pyemias OR Pyohemia OR Pyohemias OR Pyaemia OR Septicemia OR Septicemias OR Poisoning, Blood OR Poisonings, Blood OR poisoning blood OR blood poisoning OR blood poisonings OR severe sepsis OR sepsis severe OR Bacteremia OR Fungemia) [ConditionSearch]AND [InterventionSearch] oxiris  The search was conducted from the establishment of the library to November 9, 2022, and 16 protocols were retrieved. | 16 |
| **WHO ICTRP** | |
| Oxiris AND (Sepsis OR Systemic Inflammatory Response Syndrome OR Septic Shock OR Multiple Organ Failure OR Sepsis OR Systemic Inflammatory Response Syndrome OR SIRS OR Septic OR Toxic Shock OR Endotoxic Shock OR Endotoxin Shock OR Endotoxin Shocks OR (Inflammatory Response Syndrome, Systemic) OR Multiple Organ Dysfunction Syndrome OR Multiple Organ Failure OR Multiple Organ Failures OR MODS OR bloodstream infection OR bloodstream infections OR infection bloodstream OR Pyemia OR Pyemias OR Pyohemia OR Pyohemias OR Pyaemia OR Septicemia OR Septicemias OR (Poisoning, Blood) OR (Poisonings, Blood) OR poisoning blood OR blood poisoning OR blood poisonings OR severe sepsis OR sepsis severe OR Bacteremia OR Fungemia)  The search was conducted from the establishment of the library to November 9, 2022, and 21 protocols were retrieved. | 21 |
| **China National Knowledge Infrastructure^a^** | |
| (主题=脓毒血症 + 脓毒症休克 + 全身炎症反应综合征 ＋ 多器官功能障碍综合症 (精确) ) OR (篇关摘=脓毒血症 ＋ 脓毒症休克 + 全身炎症反应综合征 ＋ 多器官功能障碍综合症 ＋ 脓毒症 + 脓血症 ＋ 毒血症 + 败血症 + 菌血症 ＋ 真菌血症 + 脓毒性休克 ＋ 脓毒症性休克 ＋ 中毒性休克 + 感染性休克 ＋ 内毒素性休克 (精确) ) OR (篇关摘=全身炎症反应综合征 + SIRS ＋ 系统性炎性反应综合征 + 全身性炎性反应综合征 ＋ 系统性炎症友应综合征 ＋ 全身炎症反应综合症 ＋ 全身性炎症反应综合征 + 全身炎性反应综合征 ＋ 多器官功能不全综合征 + mods (精确) ) OR (篇关摘：多脏器功能衷竭综合征 ＋ 多脏器功能障碍综合征 ＋ 多器官功能衰竭综合征 ＋ 多脏器功能失常综合征 + 多胜器功能不全综合征 ＋ 多器官功能障碍综合征 ＋ 多脏器功能障碍 ＋ 多脏器功能障碍综合症 (精确) ) AND (全文=oxiris + 百希瑞 (精确) )  The first search was conducted from the establishment of the library to July 2022, and 26 articles were retrieved.  A supplementary search was conducted from July 2022 to November 9, 2022, and 2 articles were retrieved. | 28 |
| **VIP Database for Chinese Technical Periodicals^a^** | |
| (((((题名或关键词=脓毒血症 OR 题名或关键词=脓毒症休克) OR 题名或关键词=全身炎症反应综合征) OR 题名或关键词=多器官功能障碍综合症) OR ((((((((((((((((((((((((((((((((任意字段=脓毒血症 OR 任意字段=脓毒症休克) OR 任意字段=全身炎症反应综合征) OR 任意字段=多器官功能障碍综合症) OR 任意字段=脓毒症) OR 任意字段=脓血症) OR 任意字段=毒血症) OR 任意字段=败血症) OR 任意字段=菌血症) OR 任意字段=真菌血症) OR 任意字段=脓毒性休克) OR 任意字段=脓毒症性休克) OR 任意字段=中毒性休克) OR 任意字段=感染性休克) OR 任意字段=内毒素性休克) OR 任意字段=全身炎症反应综合征) OR 任意字段=SIRS) OR 任意字段=系统性炎性反应综合征) OR 任意字段=全身性炎性反应综合征) OR 任意字段=系统性炎症反应综合征) OR 任意字段=全身炎症反应综合症) OR 任意字段=全身性炎症反应综合征) OR 任意字段=全身炎性反应综合征) OR 任意字段=多器官功能不全综合征) OR 任意字段=mods) OR 任意字段=多脏器功能衰竭综合征) OR 任意字段=多脏器功能障碍综合征) OR 任意字段=多器官功能衰竭综合征) OR 任意字段=多脏器功能失常综合征) OR 任意字段=多脏器功能不全综合征) OR 任意字段=多器官功能障碍综合征) OR 任意字段=多脏器功能障碍) OR 任意字段=多脏器功能障碍综合症)) AND (任意字段=oxiris OR 任意字段=百希瑞))  The first search was conducted from the establishment of the library to July 2022, and 9 articles were retrieved.  A supplementary search was conducted from July 2022 to November 9, 2022, and 2 articles were retrieved. | 11 |
| **Wanfang Database^a^** | |
| (全部:(脓毒血症 OR 脓毒症休克 OR 全身炎症反应综合征 OR 多器官功能障碍综合症 OR 脓毒症 OR 脓血症 OR 毒血症 OR 菌血症) or 全部:(败血症 OR 脓毒性休克 OR 脓毒症性休克 OR 败血性休克 OR 中毒性休克 OR 感染性休克 OR 内毒素性休克) or 全部:(SIRS OR 系统性炎性反应综合征 OR 全身性炎性反应综合征 OR 系统性炎症反应综合征 OR 全身炎症反应综合症 OR 全身性炎症反应综合征 OR 全身炎性反应综合征) or 全部:(多器官功能不全综合征 OR MODS OR 多脏器功能衰竭综合征 OR 多脏器功能障碍综合征 OR 多器官功能衰竭综合征 OR 多脏器功能失常综合征) or 全部:(多脏器功能不全综合征 OR 多器官功能障碍综合征 OR 多脏器功能障碍 OR 多脏器功能障碍综合症) )AND 全部:(oXiris OR 百希瑞)  [智能检索]主题词扩展  The first search was conducted from the establishment of the library to July 2022, and 28 articles were retrieved.  A supplementary search was conducted from July 2022 to November 9, 2022, and 5 articles were retrieved. | 33 |
| **SinoMed^a^** | |
| (("脓毒血症"[全部字段] OR "脓毒症"[全部字段] OR "血液中毒"[全部字段] OR "脓血症"[全部字段] OR "败血病"[全部字段] OR "脓毒症"[主题词]) OR "脓毒症休克"[全部字段] OR ("全身炎症反应综合征"[全部字段] OR "全身性炎性反应综合征"[全部字段] OR "脓毒症综合征"[全部字段] OR "全身炎症反应综合征"[主题词]) OR "多器官功能障碍综合症"[全部字段] OR ("脓毒症"[全部字段] OR "血液中毒"[全部字段] OR "脓血症"[全部字段] OR "脓毒血症"[全部字段] OR "败血病"[全部字段] OR "脓毒症"[主题词]) OR ("脓血症"[全部字段] OR "脓毒症"[全部字段] OR "血液中毒"[全部字段] OR "脓毒血症"[全部字段] OR "败血病"[全部字段] OR "脓毒症"[主题词]) OR ("毒血症"[全部字段] OR "毒血症"[主题词]) OR ("菌血症"[全部字段] OR "菌血症"[主题词]) OR "败血症"[全部字段] OR ("脓毒性休克"[全部字段] OR "内毒素性休克"[全部字段] OR "中毒性休克"[全部字段] OR "中毒性休克综合征"[全部字段] OR "休克, 脓毒性"[主题词]) OR "脓毒症性休克"[全部字段] OR "败血性休克"[全部字段] OR "中毒性休克"[全部字段] OR "感染性休克"[全部字段] OR "内毒素性休克"[全部字段] OR "SIRS"[全部字段] OR "系统性炎性反应综合征"[全部字段] OR "全身性炎性反应综合征"[全部字段] OR "系统性炎症反应综合征"[全部字段] OR "全身炎症反应综合症"[全部字段] OR "全身性炎症反应综合征"[全部字段] OR "全身炎性反应综合征"[全部字段] OR "多器官功能不全综合征"[全部字段] OR ("MODS"[全部字段] OR "多器官功能衰竭"[全部字段] OR "多器官功能障碍综合征"[全部字段] OR "多器官功能紊乱综合征"[全部字段] OR "多器官功能衰竭"[主题词]) OR "多脏器功能衰竭综合征"[全部字段] OR "多脏器功能障碍综合征"[全部字段] OR "多器官功能衰竭综合征"[全部字段] OR "多脏器功能失常综合征"[全部字段] OR "多脏器功能不全综合征"[全部字段] OR ("多器官功能障碍综合征"[全部字段] OR "多器官功能衰竭"[全部字段] OR "MODS"[全部字段] OR "多器官功能紊乱综合征"[全部字段] OR "多器官功能衰竭"[主题词]) OR "多脏器功能障碍"[全部字段] OR "多脏器功能障碍综合症"[全部字段]) AND ("oXiris"[全部字段] OR "百希瑞"[全部字段])  The first search was conducted from the establishment of the library to July 2022, and 9 articles were retrieved.  A supplementary search was conducted from July 2022 to November 9, 2022, and 1 articles were retrieved. | 10 |
| ( "Sepsis"[全部字段:智能] OR "Systemic Inflammatory Response Syndrome"[全部字段:智能] OR "Septic Shock"[全部字段:智能] OR "Multiple Organ Failure"[全部字段:智能] OR "Sepsis"[全部字段:智能] OR "Systemic Inflammatory Response Syndrome"[全部字段:智能] OR "SIRS"[全部字段:智能] OR "Septic"[全部字段:智能] OR "Toxic Shock"[全部字段:智能] OR "Endotoxic Shock"[全部字段:智能] OR "Endotoxin Shock"[全部字段:智能] OR "Endotoxin Shocks"[全部字段:智能] OR "(Inflammatory Response Syndrome, Systemic)"[全部字段:智能] OR "Multiple Organ Dysfunction Syndrome"[全部字段:智能] OR "Multiple Organ Failure"[全部字段:智能] OR "Multiple Organ Failures"[全部字段:智能] OR "MODS"[全部字段:智能] OR "bloodstream infection"[全部字段:智能] OR "bloodstream infections"[全部字段:智能] OR "infection bloodstream"[全部字段:智能] OR "Pyemia"[全部字段:智能] OR "Pyemias"[全部字段:智能] OR "Pyohemia"[全部字段:智能] OR "Pyohemias"[全部字段:智能] OR "Pyaemia"[全部字段:智能] OR "Septicemia"[全部字段:智能] OR "Septicemias"[全部字段:智能] OR "(Poisoning, Blood)"[全部字段:智能] OR "(Poisonings, Blood)"[全部字段:智能] OR "poisoning blood"[全部字段:智能] OR "blood poisoning"[全部字段:智能] OR "blood poisonings"[全部字段:智能] OR "severe sepsis"[全部字段:智能] OR "sepsis severe"[全部字段:智能] OR "Bacteremia"[全部字段:智能] OR "Fungemia"[全部字段:智能]) AND "oXiris"[全部字段:智能]  The first search was conducted from the establishment of the library to July 2022, and 25 articles were retrieved.  A supplementary search was conducted from July 2022 to November 9, 2022, and 3 articles were retrieved. | 28 |
| **Chinese Clinical Trial Registry^a^** | |
| 研究疾病名称：脓毒症  干预措施：oxiris  The search was conducted from the establishment of the library to November 9, 2022, and 4 protocols were retrieved. | 4 |

1. In order to more accurately show the details of the search and facilitate the reproduction of searching process, the search query of the Chinese database is described in Chinese.

Appendix 2: Characteristics of different filters

AN69 membranes are commonly used synthetic membranes in blood purification therapy, effectively removing urinary toxins and cytokines from plasma [[1](#_ENREF_1)] . Based on the AN69 membrane, engineers further optimized and developed the AN69-ST membrane and oXiris membrane. AN69-ST membrane is a polyacrylonitrile membrane coated with polyethylenimine and heparin, which can reduce the adsorption of large molecular proteins but has no effect on the adsorption of low and medium molecular proteins and can reduce the anticoagulant demand when used in patients with a high risk of bleeding. oXiris membrane contains three times the polyvinylimide surface coating compared to the AN69-ST membrane. It can also adsorb negatively charged endotoxins via ionic bonds while maintaining the adsorption properties of the AN69-ST membrane [[2](#_ENREF_2)] . FX80 film has good biocompatibility, and the FX80 filter is a high-flux dialyzer with a higher clearance rate of medium and large molecular toxins than ordinary filters [[3](#_ENREF_3), [4](#_ENREF_4)].

Supplementary Table 1 Characteristics of different filters.

| Filter name | Sorbent type | Toxins/mediators removed | Membrane area(m^2^) |
| --- | --- | --- | --- |
| oXiris filter | AN69 with PEI surface treatment;  endotoxin adsorbed by means of ionic interactions at the membrane surface | Uremic toxins, endotoxin, cytokines | 1.5 |
| AN69-ST100 filter | AN69-ST copolymer membrane | Uremic toxins, cytokines | 1.0 |
| AN69-ST150 filter |  |  | 1.5 |
| M100 filter | AN69 membrane | Uremic toxins, cytokines | 1.0 |
| M150 filter |  |  | 1.5 |
| FX80 filter | Polysulfone membrane | Uremic toxins, cytokines | 1.8 |

Appendix 3: Risk of bias assessment

Supplementary Table 2 Quality evaluation results of NOS included in the study

| **Study** | **Selection** | | | |  | **Comparability** |  | **Outcome** | | |  | **Total score*** |
| --- | --- | --- | --- | --- | --- | --- | --- | --- | --- | --- | --- | --- |
|  | **Represent­ativeness of the exposed cohort** | **Selection of the non exposed cohort** | **Ascertain­ment of exposure** | **Demonstration that outcome of interest was not present at start of study** |  | **Comparability of cohorts on the basis of the design or analysis** |  | **Assess­ment of outcome** | **Was follow-up long enough for outcomes to occur** | **Adequacy of follow up of cohorts** |  |  |
| Zang 2020 [[5](#_ENREF_5)] | 1 | 1 | 1 | 0 |  | 2 |  | 1 | 1 | 1 |  | 8 |
| Shum 2013 [[6](#_ENREF_6)] | 1 | 0 | 1 | 0 |  | 2 |  | 1 | 1 | 1 |  | 7 |
| Zang 2022 [[7](#_ENREF_7)] | 1 | 1 | 1 | 1 |  | 2 |  | 1 | 1 | 1 |  | 9 |
| Guan 2022 [[8](#_ENREF_8)] | 1 | 1 | 1 | 0 |  | 0 |  | 1 | 1 | 1 |  | 6 |
| Xie 2022 [[9](#_ENREF_9)] | 1 | 1 | 1 | 0 |  | 2 |  | 1 | 1 | 1 |  | 8 |
| Zhai 2021 [[10](#_ENREF_10)] | 1 | 1 | 1 | 1 |  | 2 |  | 1 | 1 | 1 |  | 9 |
| Le 2022 [[11](#_ENREF_11)] | 1 | 1 | 1 | 0 |  | 2 |  | 1 | 1 | 1 |  | 8 |
| Yu 2020 [[12](#_ENREF_12)] | 1 | 0 | 1 | 0 |  | 2 |  | 1 | 1 | 1 |  | 7 |
| Lin 2021 [[13](#_ENREF_13)] | 1 | 1 | 1 | 0 |  | 2 |  | 1 | 1 | 1 |  | 8 |
| Kang 2022 [[14](#_ENREF_14)] | 1 | 1 | 1 | 0 |  | 2 |  | 1 | 1 | 1 |  | 8 |

*: Maximum score is 9. Scores <6 were classified as low-quality studies, 6 to 7 as intermediate-quality studies, and 8 to 9 as high-quality studies.

Appendix 4: Forest plots of outcomes adopting Xie’s data before IPTW


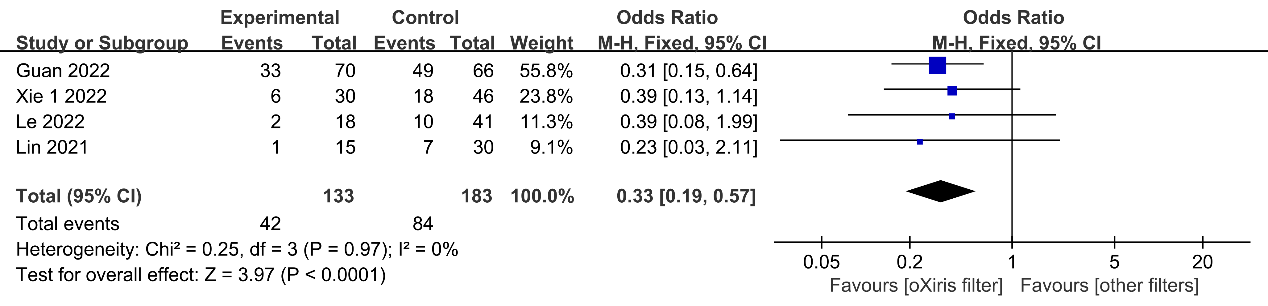


Fig. S1 7-day mortality (adopting Xie’s data before IPTW)


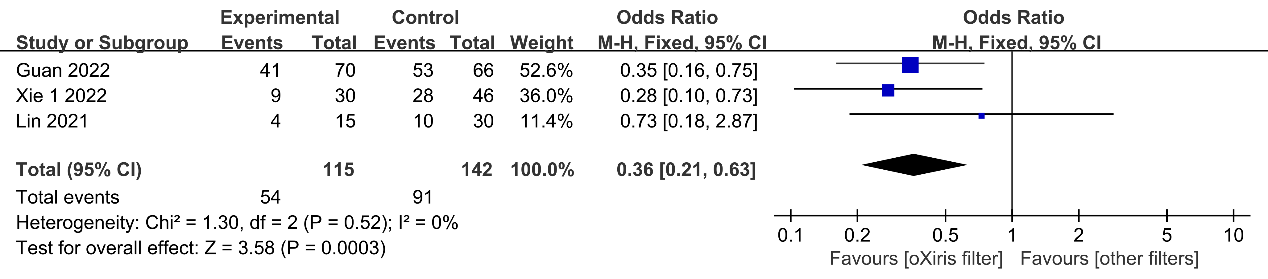


Fig. S2 14-day mortality (adopting Xie’s data before IPTW)


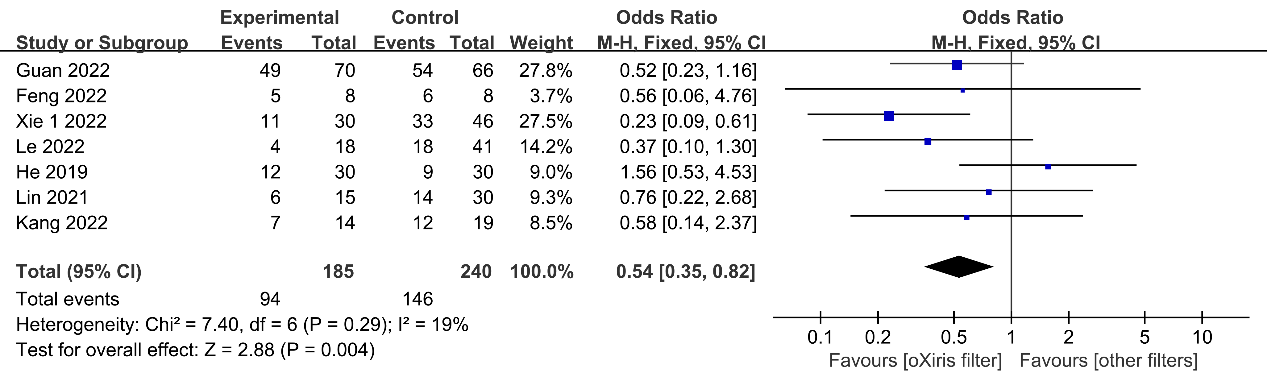


Fig. S3 28-day mortality (adopting Xie’s data before IPTW)


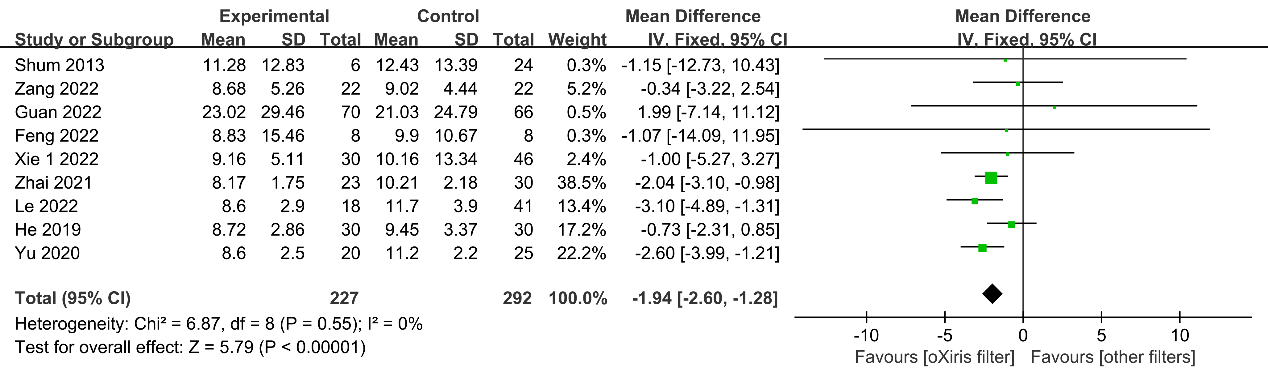


Fig. S4 the length of ICU stay (adopting Xie’s data before IPTW)


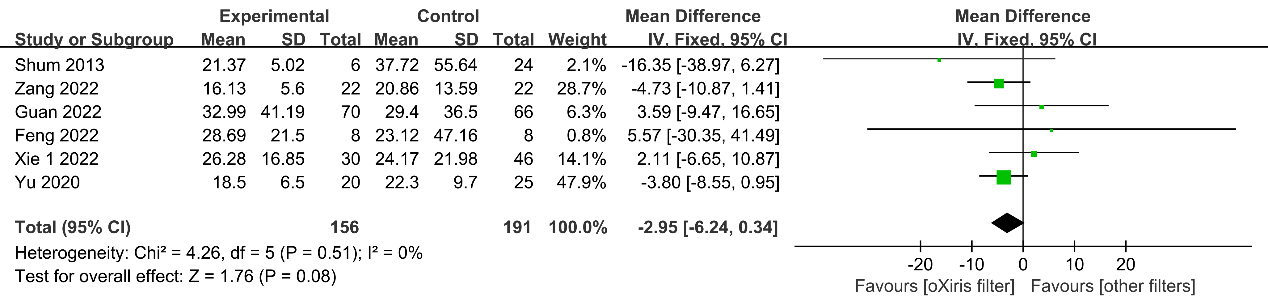


Fig. S5 the length of hospital stay (adopting Xie’s data before IPTW)

Appendix 5: Results of secondary outcomes

The pooled meta-analysis results showed that the oXiris filter was associated with significantly lower mortality in patients with sepsis compared to other filters at seven days [adopting Xie’s data before inverse probability treatment weighting (IPTW): fixed-effect model, odds ratio (OR) 0.33; 95% confidence interval (CI) 0.19-0.57, P < 0.001, I^2^ = 0%; Fig. S1; adopting Xie’s data after IPTW: OR 0.41; 95% CI 0.26-0.66, P < 0.001, I^2^ = 0%; Fig. S6] and 14 days (adopting Xie’s data before IPTW: OR 0.36; 95% CI 0.21-0.63, P < 0.001, I^2^ = 0%; Fig. S2; adopting Xie’s data after IPTW: OR 0.42; 95% CI 0.26-0.67, P < 0.001, I^2^ = 0%; Fig. S7). Besides, no significant heterogeneity was found in the studies with the 7- and 14-day mortalities.

However, there was no statistical difference in the length of hospital stay (adopting Xie’s data before IPTW: weighted mean difference (WMD) −2.95; 95% CI −6.24 to 0.34, P = 0.08, I^2^ = 0%; Fig. S5; adopting Xie’s data after IPTW: WMD −3.02; 95% CI −6.25 to 0.21, P = 0.07, I^2^ = 0%; Fig. S8). No significant heterogeneity was found among the studies.

Besides, there was no statistical difference in the 90-day mortality (OR 0.54; 95% CI 0.28-1.04, P = 0.06, I^2^ = 0%; Fig. S9), ICU mortality (OR 0.59; 95% CI 0.19-1.78, P = 0.35, I^2^ = 0%; Fig. S10), and hospital mortality (OR 1.04; 95% CI 0.59-1.85, P = 0.89, I^2^ = 0%; Fig. S11) between the two groups. No significant heterogeneity was found among the studies.

All outcomes of the meta-analysis are summarized in Supplementary Table 3.

**
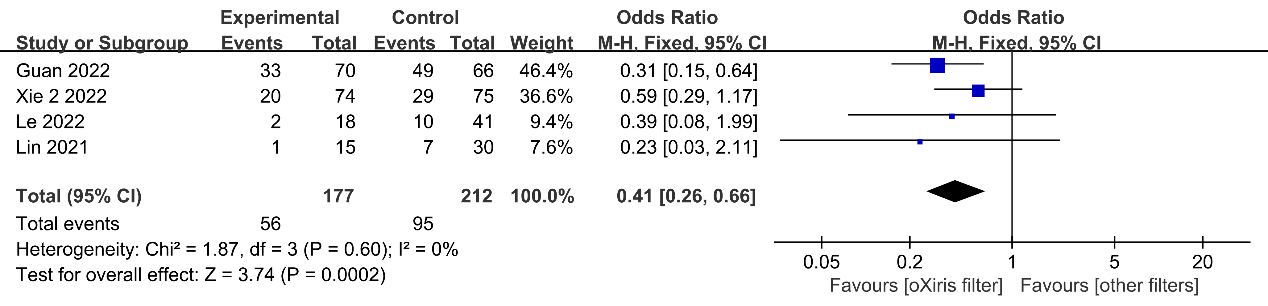
**

Fig. S6 7-day mortality (adopting Xie’s data after IPTW)


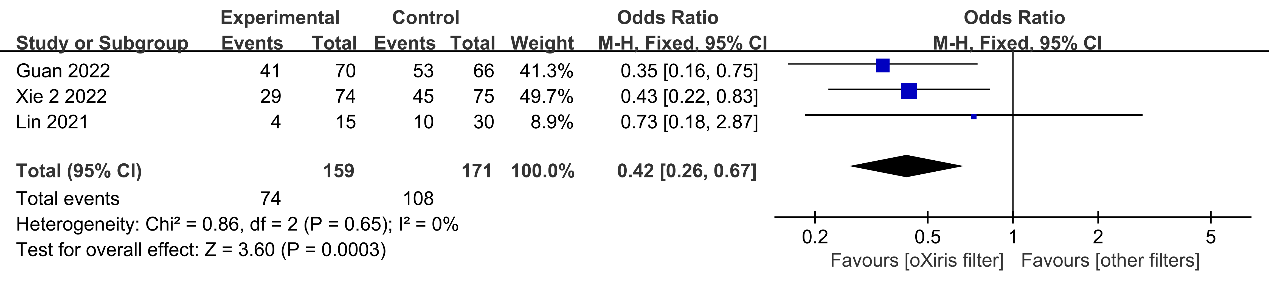


Fig. S7 14-day mortality (adopting Xie’s data after IPTW)


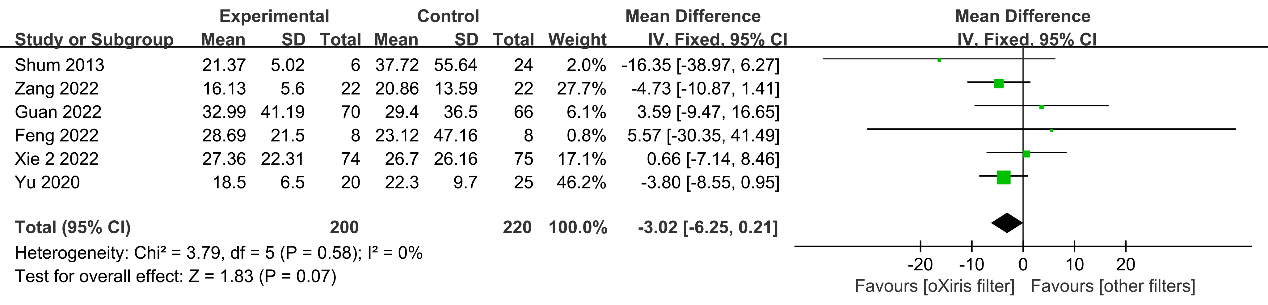


Fig. S8 the length of hospital stay (adopting Xie’s data after IPTW)


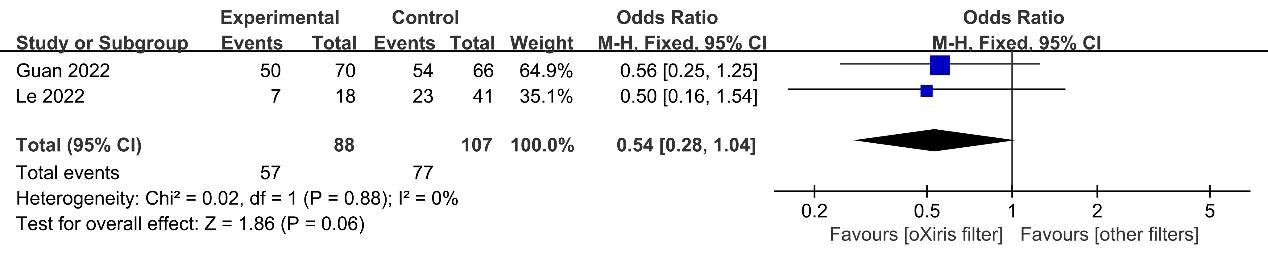


Fig. S9 90-day mortality


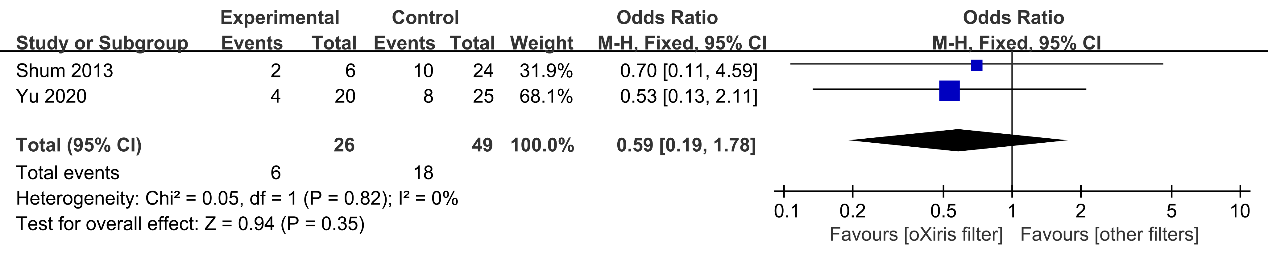


Fig. S10 ICU mortality


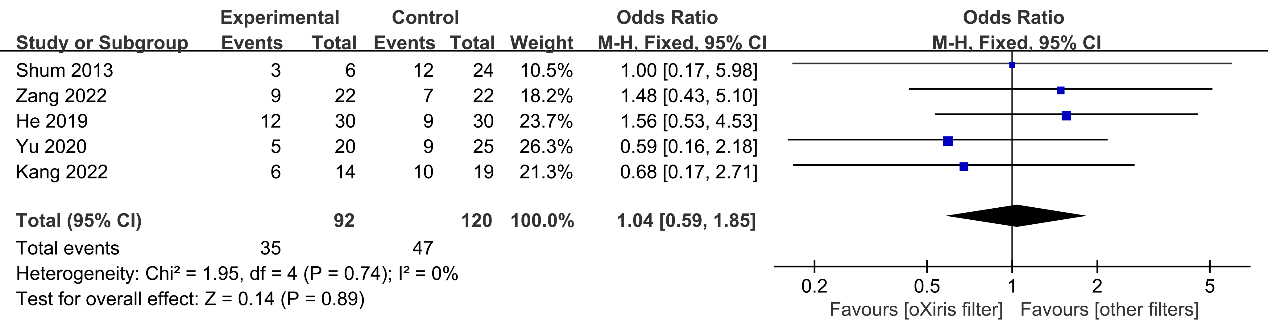


Fig. S11 hospital mortality

Supplementary Table 3 outcomes of the meta-analysis

| **Outcome** | **n** | **WMD/OR** | **95% CI** | **p-value** | **Heterogeneity** | |
| --- | --- | --- | --- | --- | --- | --- |
| Binary variable (OR) | | | | | | |
| 7d Mortality^a^ | 316 | 0.33 | (0.19, 0.57) | **＜0.01** | I²=0% | P=0.97 |
| 7d Mortality^b^ | 389 | 0.41 | (0.26, 0.66) | **＜0.01** | I²=0% | P=0.60 |
| 14d Mortality^a^ | 257 | 0.36 | (0.21, 0.63) | **＜0.01** | I²=0% | P=0.52 |
| 14d Mortality^b^ | 330 | 0.42 | (0.26, 0.67) | **＜0.01** | I²=0% | P=0.65 |
| 28d Mortality^a^ | 392 | 0.54 | (0.35, 0.82) | **＜0.01** | I²=19% | P=0.19 |
| 28d Mortality^b^ | 498 | 0.53 | (0.36, 0.77) | **＜0.01** | I²=8% | P=0.37 |
| 90d Mortality | 195 | 0.54 | (0.28, 1.04) | 0.06 | I²=0% | P=0.88 |
| ICU Mortality | 75 | 0.59 | (0.19, 1.78) | 0.35 | I²=0% | P=0.82 |
| Hospital Mortality | 212 | 1.04 | (0.59, 1.85 ) | 0.89 | I²=0% | P=0.74 |
|  | | | | | | |
| Continuous variable (WMD) | | | | | | |
| length of ICU stay^a^ | 519 | -1.94 | (-2.60, -1.28) | **＜0.01** | I²=0% | P=0.55 |
| length of ICU stay^b^ | 592 | -1.91 | (-2.56, -1.26) | **＜0.01** | I²=0% | P=0.50 |
| length of hospital stay^a^ | 347 | -2.95 | (-6.24, 0.34) | 0.08 | I²=0% | P=0.51 |
| length of hospital stay^b^ | 420 | -3.02 | (-6.25, 0.21) | 0.07 | I²=0% | P=0.58 |
| NE dose | 191 | -0.11 | (-0.17, -0.06) | **＜0.01** | I²=46% | P=0.14 |
| lactate level | 511 | -0.49 | (-0.78, -0.19) | **＜0.01** | I²=74% | P<0.01 |
| SOFA score | 347 | -1.41 | (-1.92, -0.91) | **＜0.01** | I²=50% | P=0.04 |
|  |  |  |  |  |  |  |
| Continuous variable (SMD) | | | | | | |
| IL-6 level | 235 | -0.75 | (-1.02, -0.48) | **＜0.01** | I²=16% | P=0.31 |

a: The data is before IPTW; b: The data is after IPTW.

NE: norepinephrine; SOFA: sequential organ failure assessment; IL-6: interleukin-6; SMD: standardized mean difference; WMD: weighted mean difference.

Appendix 6: Subgroup analyses of primary and secondary outcomes

Subgroup analyses were conducted according to the type of studies. The meta-analysis results of observational studies [[8](#_ENREF_8), [9](#_ENREF_9), [11](#_ENREF_11), [13](#_ENREF_13), [14](#_ENREF_14)] showed that oXiris filters were associated with significantly lower 28-day mortality in patients with sepsis compared to other filters (OR 0.44; 95% CI 0.29-0.67, P < 0.001, I^2^ = 0%; Fig. S12). The RCT [[15](#_ENREF_15), [16](#_ENREF_16)] results showed no significant difference between the oXiris and control groups (OR 1.26; 95% CI 0.49-3.25, P = 0.63, I^2^ = 0%; Fig. S12). For the hospital mortality, the meta-analysis results of observational studies [[6](#_ENREF_6), [7](#_ENREF_7), [12](#_ENREF_12), [14](#_ENREF_14)] and RCT [[16](#_ENREF_16)] showed no significant difference between the oXiris and control groups (observational studies: OR 0.88; 95% CI 0.45-1.76, P = 0.72, I^2^ = 0%; Fig. S13; RCTs: OR 1.56; 95% CI 0.53-4.53; P = 0.42; Fig. S13). The meta-analysis results of observational studies [[6-12](#_ENREF_6)] showed that oXiris filters were associated with significantly shorter length of ICU stay in patients with sepsis compared to other filters [WMD −2.15; 95% CI −2.87 to −1.44, P < 0.001, I^2^ = 0%; Fig. S14], and the RCT [[15](#_ENREF_15), [16](#_ENREF_16)] results showed no significant difference between the oXiris and control groups (WMD −0.73; 95% CI −2.31 to 0.84, P = 0.36, I^2^ = 0%; Fig. S14). For the length of hospital stay, the meta-analysis results of observational studies [[6-9](#_ENREF_6), [12](#_ENREF_12)] and RCT [[15](#_ENREF_15)] showed no significant difference between the oXiris and control groups (observational studies: WMD −3.09; 95% CI −6.33 to 0.15, P = 0.06, I^2^ = 0%; Fig. S15; RCTs: WMD 5.57; 95% CI −30.35 to 41.49, P = 0.76; Fig. S15). The meta-analysis results of observational studies [[5-7](#_ENREF_5), [10](#_ENREF_10), [12-14](#_ENREF_12)] showed that oXiris filters were associated with significantly lower SOFA score in patients with sepsis compared to other filters (WMD −1.71; 95% CI −2.51 to −0.90, P < 0.001, I^2^ = 31%; Fig. S16), and the RCT [[2](#_ENREF_2), [17](#_ENREF_17)] results showed no significant difference between the oXiris and control groups (WMD 0.36; 95% CI −3.46 to 4.17, P = 0.86, I^2^ = 82%; Fig. S16). The meta-analysis results of observational studies [[5](#_ENREF_5), [7](#_ENREF_7), [9](#_ENREF_9), [10](#_ENREF_10), [12-14](#_ENREF_12)] showed that oXiris filters were associated with significantly lower lactate level in patients with sepsis compared to other filters (WMD −0.48; 95% CI −0.83 to −0.14, P < 0.01, I^2^ = 79%; Fig. S17), and the RCT [[16](#_ENREF_16), [17](#_ENREF_17)] results showed no significant difference between the oXiris and control groups (WMD −0.51; 95% CI −1.15 to 0.12, P = 0.11, I^2^ = 59%; Fig. S17). The meta-analysis results of observational studies [[10](#_ENREF_10), [12](#_ENREF_12), [14](#_ENREF_14)] showed that oXiris filters were associated with significantly lower NE dose in patients with sepsis compared to other filters (WMD −0.13; 95% CI −0.22 to −0.04, P < 0.01, I^2^ = 62%; Fig. S18), and the RCT [[17](#_ENREF_17)] results showed no significant difference between the oXiris and control groups (WMD −0.20; 95% CI −0.54 to 0.14, P = 0.25; Fig. S18). The meta-analysis results of observational studies [[7](#_ENREF_7), [10](#_ENREF_10), [13](#_ENREF_13), [14](#_ENREF_14)] and RCT [[16](#_ENREF_16)] showed that oXiris filters were associated with significantly lower IL-6 level in patients with sepsis compared to other filters (observational studies: WMD −0.77; 95% CI −1.09 to −0.46, P < 0.001, I^2^ = 36%; Fig. S19; RCT: WMD −0.69; 95% CI −1.21 to −0.16, P = 0.01; Fig. S19).


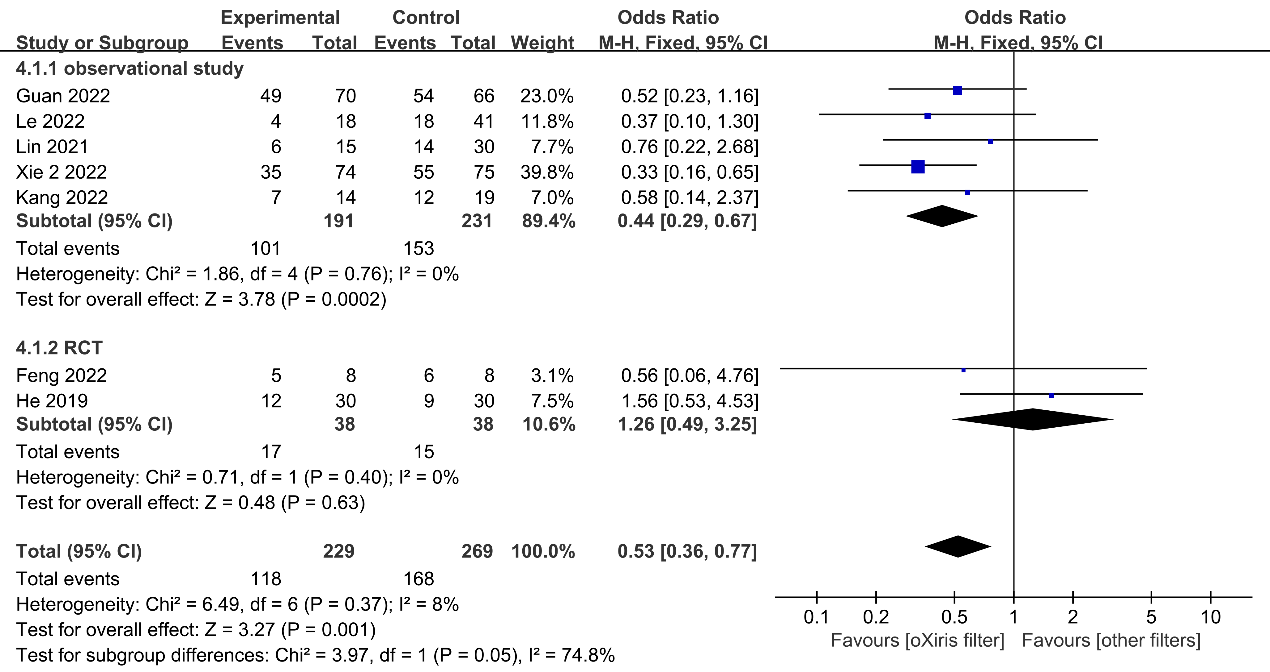


Fig. S12 subgroup analysis of 28-day mortality (adopting Xie’s data after IPTW)


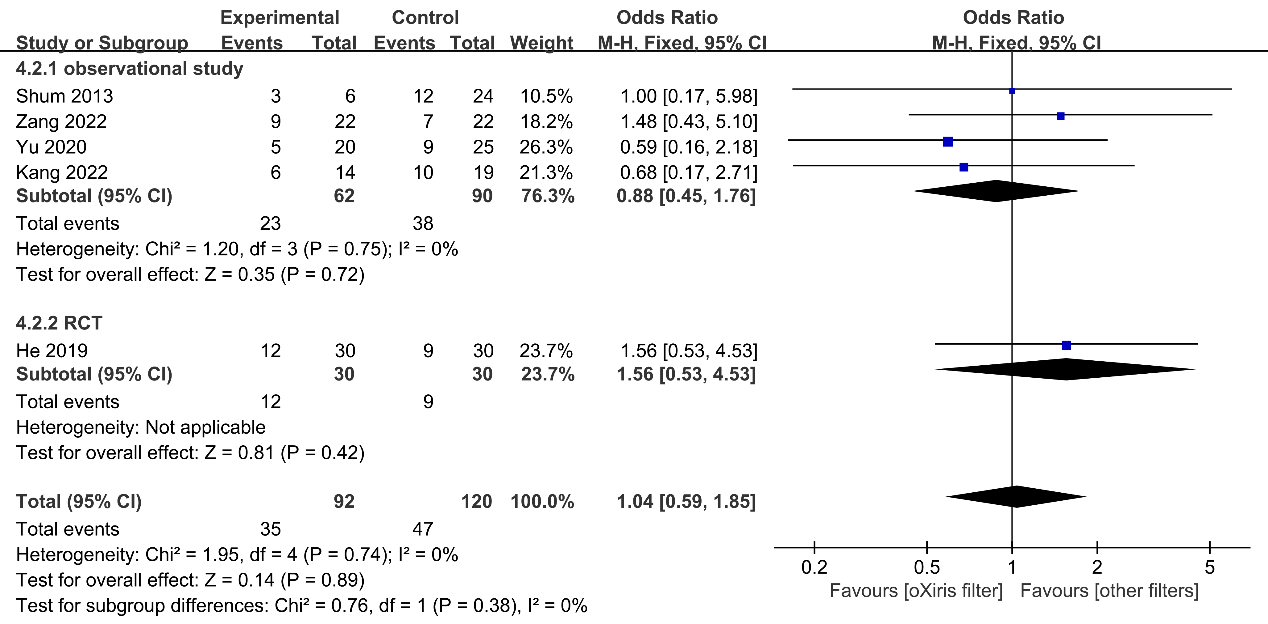


Fig. S13 subgroup analysis of hospital mortality


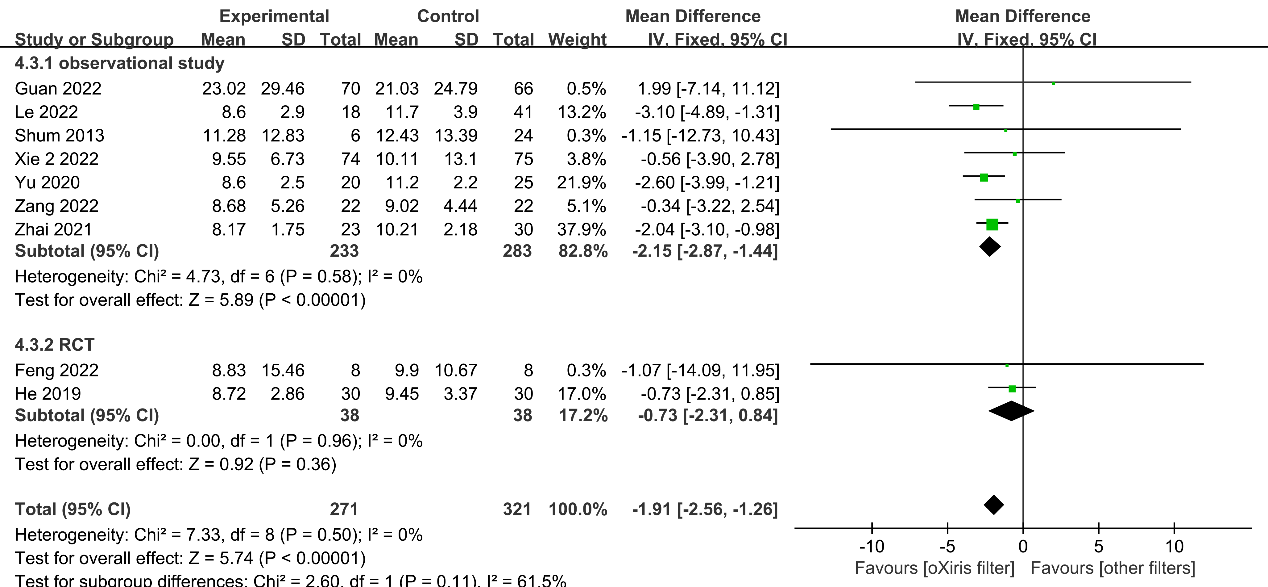


Fig. S14 subgroup analysis of the length of ICU stay (adopting Xie’s data after IPTW)


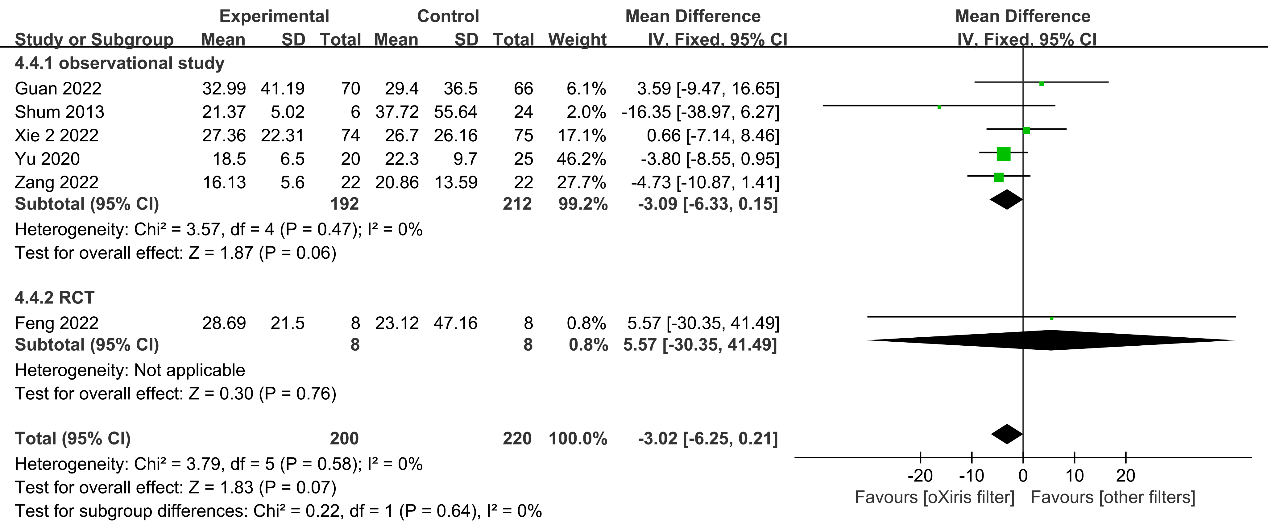


Fig. S15 subgroup analysis of the length of hospital stay (adopting Xie’s data after IPTW)


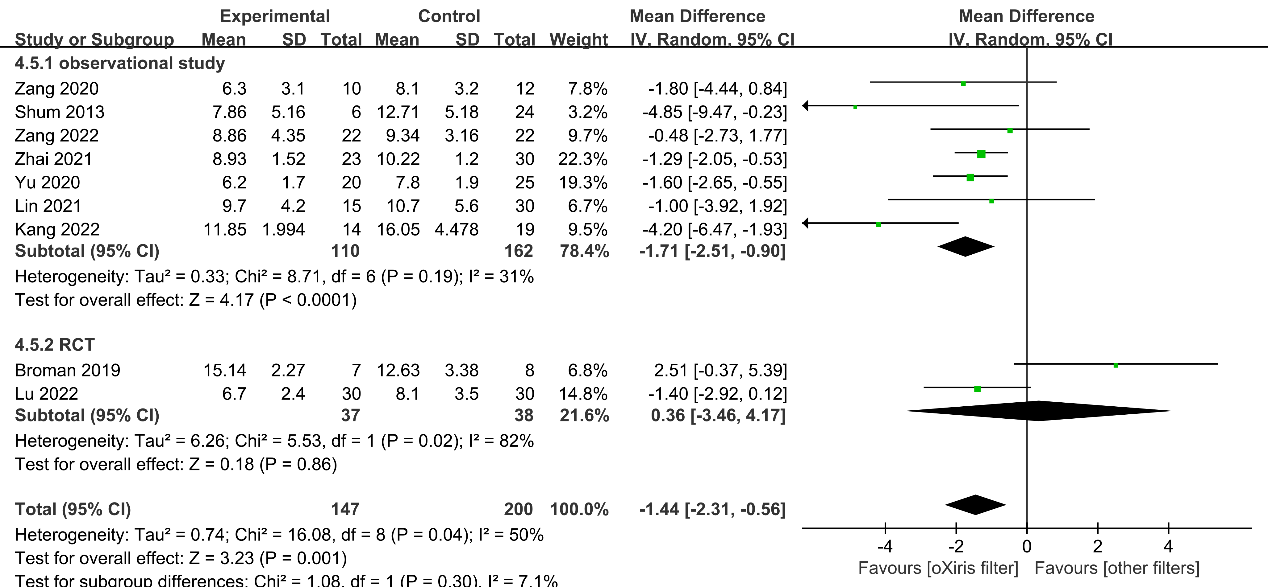


Fig. S16 subgroup analysis of the SOFA score


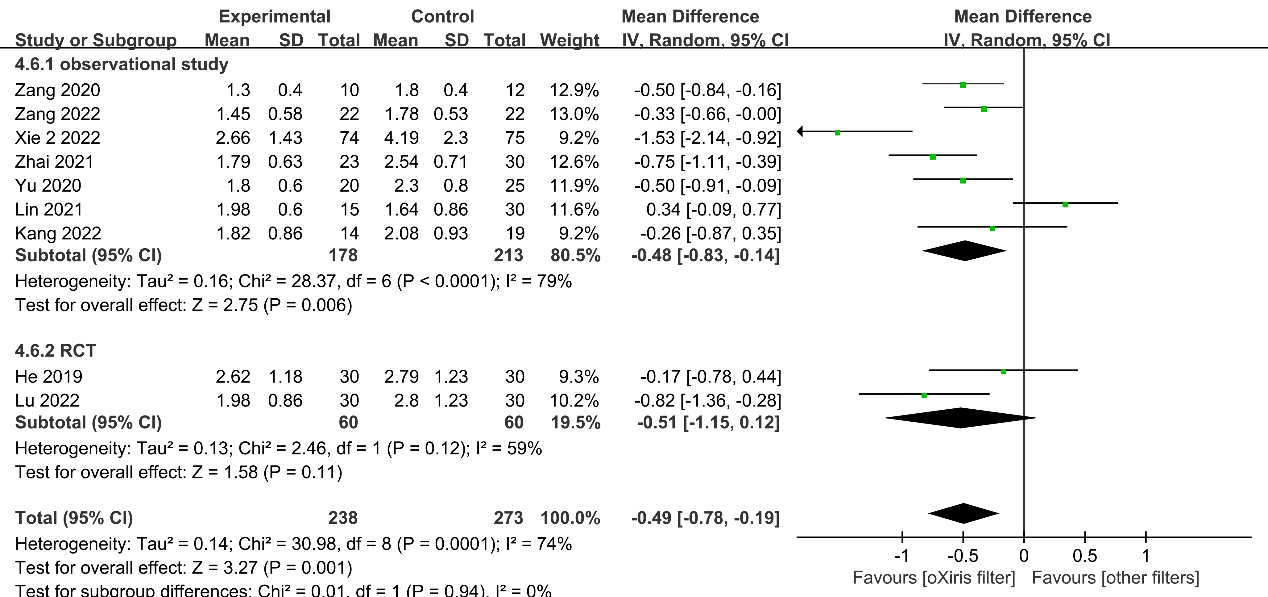


Fig. S17 subgroup analysis of the lactate level


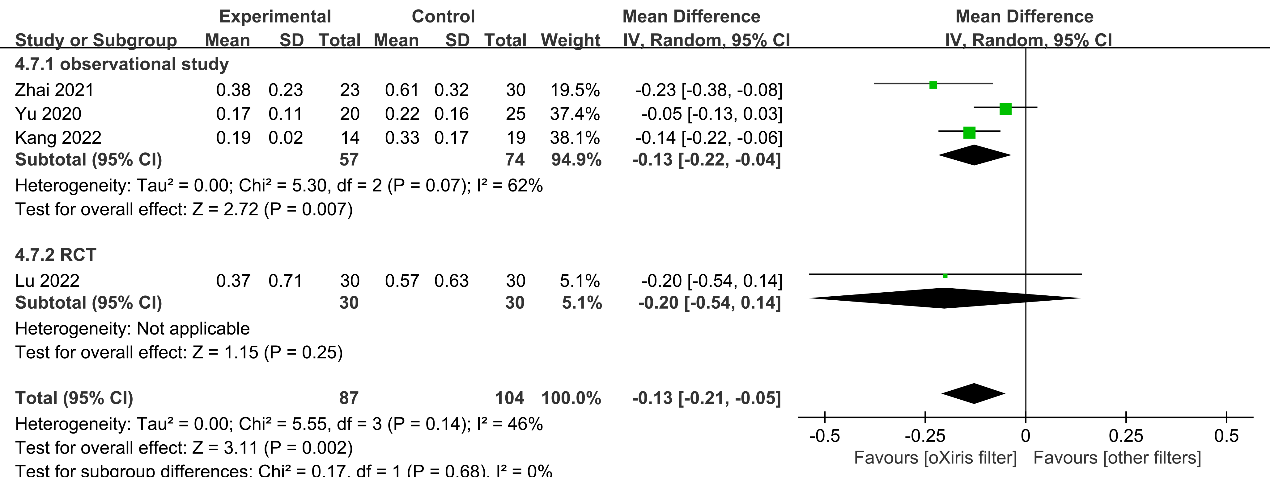


Fig. S18 subgroup analysis of the norepinephrine dose


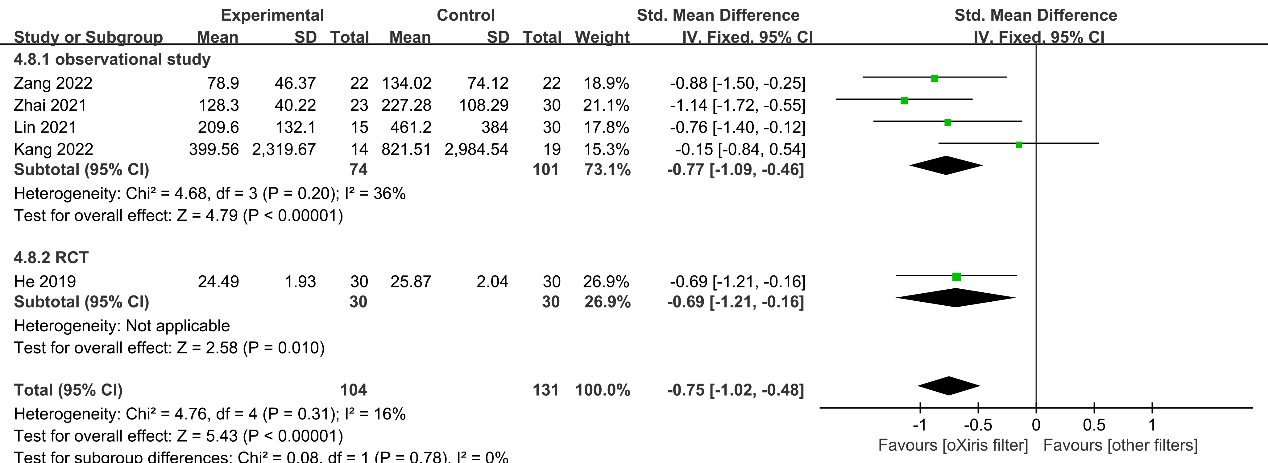


Fig. S19 subgroup analysis of the IL-6 level

Appendix 7: Sensitivity analyses by excluding low-quality studies

In He’s and Lu’s studies [[16](#_ENREF_16), [17](#_ENREF_17)], most terms of the Cochrane Risk of Bias Tool were rated as unclear risk of bias. Therefore, we excluded these studies for sensitivity analysis. Regardless of whether He’s and Lu’s studies [[16](#_ENREF_16), [17](#_ENREF_17)] were excluded, the meta-analyses results showed that oXiris filters were associated with lower 28-day mortality, SOFA score, lactate level, NE dose, IL-6 level, and shorter length of ICU stay, but not with lower hospital mortality, as shown in Figs. 3 to 8 in the body of the article and Fig. S20-Fig. S26 and Fig. S11 in this additional file.


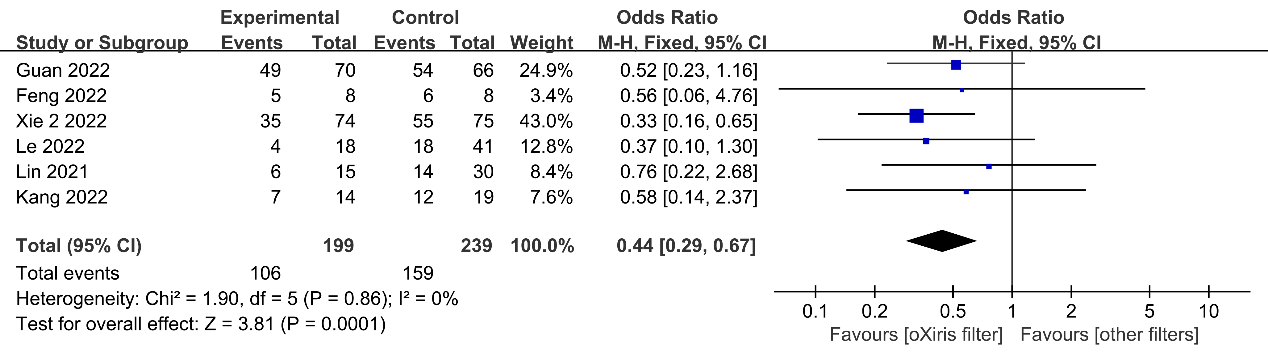


Fig. S20 28-day mortality (adopting Xie’s data after IPTW; excluding He’s study)


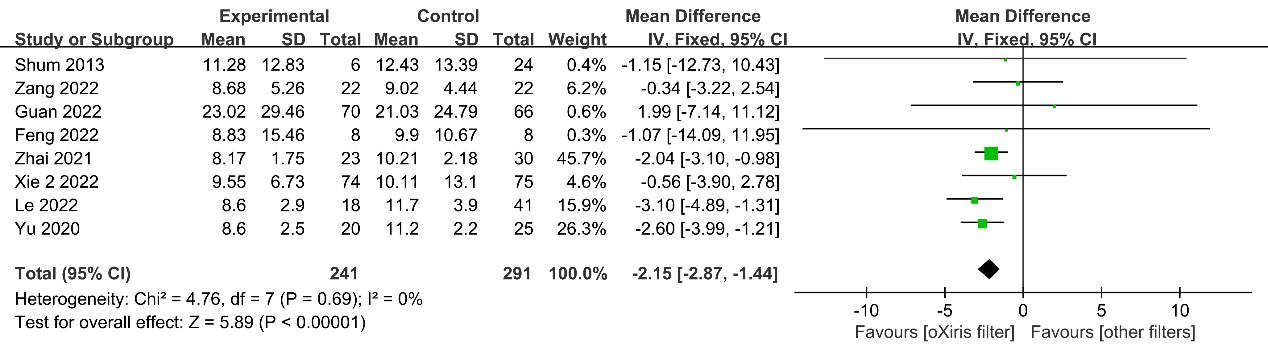


Fig. S21 the length of ICU stay (adopting Xie’s data after IPTW; excluding He’s study)


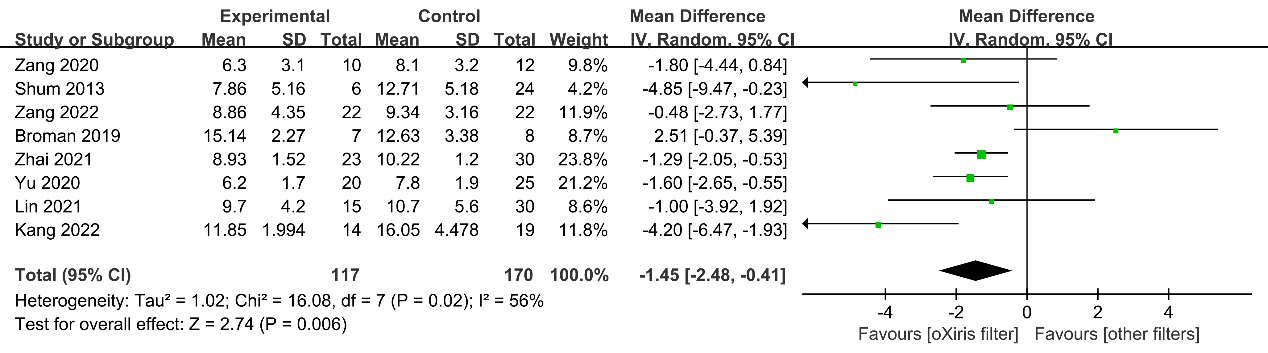


Fig. S22 SOFA score (excluding Lu’s study)


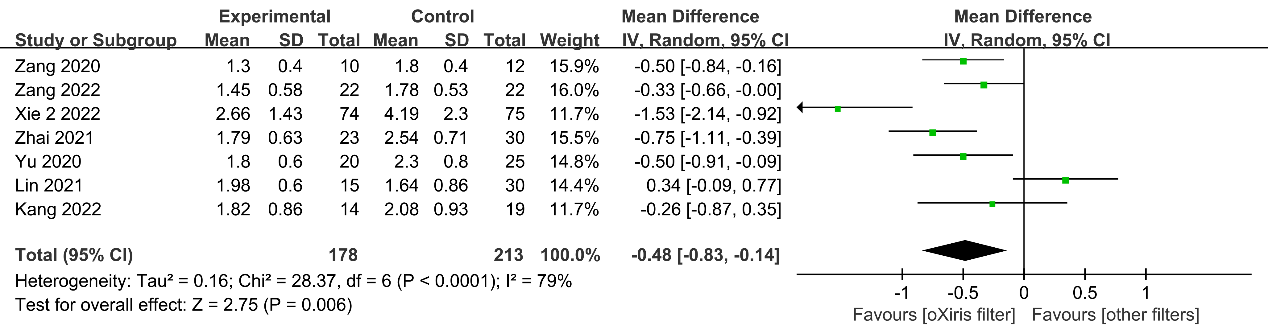


Fig. S23 lactate level (excluding He's and Lu's studies)


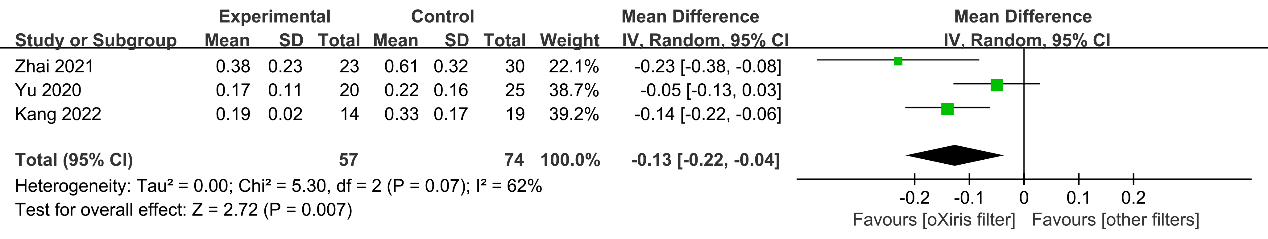


Fig. S24 norepinephrine dose (excluding Lu’s study)


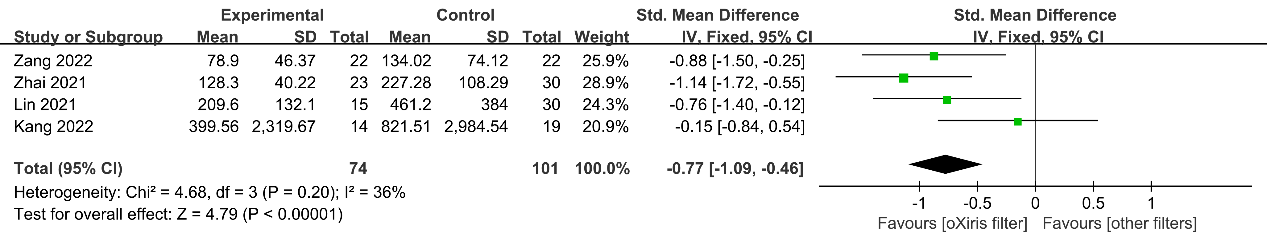


Fig. S25 IL-6 level (excluding He’s study)


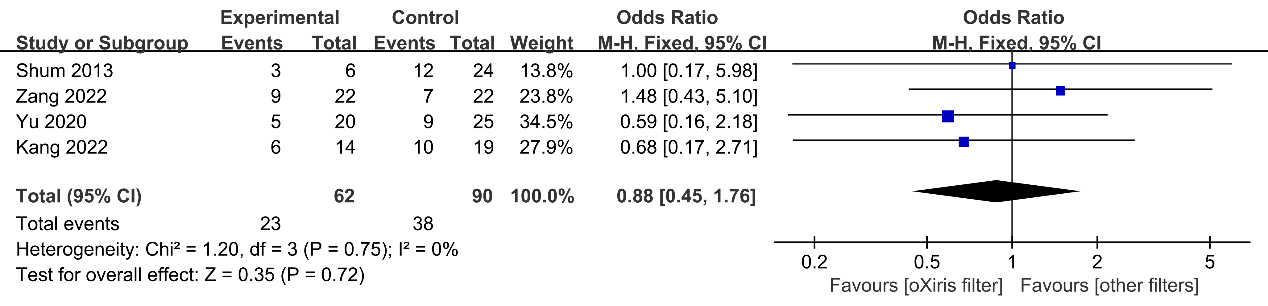


Fig. S26 hospital mortality (excluding He’s study)

Appendix 8: Certainty of evidence

Supplementary Table 4 Certainty of evidence of outcomes

| **Certainty assessment** | | | | | | | | **Number of patients** | | **Effect** | | **Certainty** | **Importance** |
| --- | --- | --- | --- | --- | --- | --- | --- | --- | --- | --- | --- | --- | --- |
| **outcome** | **Number of studies** | **Study design** | **Risk of bias** | **Incon­sistency** | **Indi­rectness** | **Impre­cision** | **Other con­siderations** | **oXiris** | **other filters** | **Relative**  **(95% CI)** | **Absolute**  **(95% CI)** |  |  |
| **7-day mortality** | 4 | observational studies | not serious | not serious | not serious | not serious | none | 56/177 (31.6%) | 95/212 (44.8%) | **OR 0.41**  (0.26 to 0.66) | **198 fewer per 1,000**  (from 274 fewer to 99 fewer) | ⨁⨁◯◯  Low | CRITICAL |
| **14-day mortality** | 3 | observational studies | not serious | not serious | not serious | not serious | none | 74/159 (46.5%) | 108/171 (63.2%) | **OR 0.42**  (0.26 to 0.67) | **213 fewer per 1,000**  (from 323 fewer to 97 fewer) | ⨁⨁◯◯  Low | CRITICAL |
| **28-day mortality^i^** | 2 | randomized trials | serious^a^ | not serious | not serious | very serious^e^ | publication bias strongly suspected^c^ | 17/38 (44.7%) | 15/38 (39.5%) | **OR 1.26**  (0.49 to 3.25) | **56 more per 1,000**  (from 153 fewer to 285 more) | ⨁◯◯◯  Very low | CRITICAL |
| **28-day mortality^j^** | 5 | observational studies | not serious | not serious | not serious | not serious | none | 101/191 (52.9%) | 153/231 (66.2%) | **OR 0.44**  (0.29 to 0.67) | **199 fewer per 1,000**  (from 300 fewer to 94 fewer) | ⨁⨁◯◯  Low | CRITICAL |
| **90-day mortality** | 2 | observational studies | not serious | not serious | not serious | very serious^d^ | none | 57/88 (64.8%) | 77/107 (72.0%) | **OR 0.54**  (0.28 to 1.04) | **139 fewer per 1,000**  (from 301 fewer to 8 more) | ⨁◯◯◯  Very low | CRITICAL |
| **ICU mortality** | 2 | observational studies | not serious | not serious | not serious | very serious^d^ | publication bias strongly suspected^c^ | 6/26 (23.1%) | 18/49 (36.7%) | **OR 0.59**  (0.19 to 1.78) | **112 fewer per 1,000**  (from 268 fewer to 141 more) | ⨁◯◯◯  Very low | CRITICAL |
| **hospital mortality^i^** | 1 | randomized trials | serious^a^ | not serious | not serious | very serious^e^ | publication bias strongly suspected^c^ | 12/30 (40.0%) | 9/30 (30.0%) | **OR 1.56**  (0.53 to 4.53) | **101 more per 1,000**  (from 115 fewer to 360 more) | ⨁◯◯◯  Very low | CRITICAL |
| **hospital mortality^j^** | 4 | observational studies | not serious | not serious | not serious | very serious^d^ | none | 17/48 (35.4%) | 28/71 (39.4%) | **OR 0.88**  (0.45 to 1.76) | **31 fewer per 1,000**  (from 175 fewer to 140 more) | ⨁◯◯◯  Very low | CRITICAL |
| **length of ICU stay^i^** | 2 | randomized trials | serious^a^ | not serious | not serious | very serious^d^ | publication bias strongly suspected^c^ | 38 | 38 | - | WMD **0.73 d fewer**  (2.31 fewer to 0.84 more) | ⨁◯◯◯  Very low | IMPORTANT |
| **length of ICU stay^j^** | 7 | observational studies | not serious | not serious | not serious | not serious | none | 233 | 283 | - | WMD **2.15 d fewer**  (2.87 fewer to 1.44 fewer) | ⨁⨁◯◯  Low | IMPORTANT |
| **length of hospital stay^i^** | 1 | randomized trials | not serious | not serious | not serious | very serious^e^ | publication bias strongly suspected^c^ | 8 | 8 | - | WMD **5.57 d more**  (30.35 fewer to 41.49 more) | ⨁◯◯◯  Very low | IMPORTANT |
| **length of hospital stay^j^** | 5 | observational studies | not serious | not serious | not serious | serious^f^ | none | 192 | 212 | - | WMD **3.09 d fewer**  (6.33 fewer to 0.15 more) | ⨁◯◯◯  Very low | IMPORTANT |
| **NE dose^i^** | 1 | randomized trials | serious^a^ | not serious | not serious | very serious^d^ | publication bias strongly suspected^c^ | 30 | 30 | - | MD **0.2 μg/kg/min fewer**  (0.54 fewer to 0.14 more) | ⨁◯◯◯  Very low | IMPORTANT |
| **NE dose^j^** | 3 | observational studies | not serious | serious^g^ | not serious | serious^b^ | none | 57 | 74 | - | MD **0.13 μg/kg/min fewer**  (0.22 fewer to 0.04 fewer) | ⨁◯◯◯  Very low | IMPORTANT |
| **SOFA score^i^** | 2 | randomized trials | serious^a^ | very serious^h^ | not serious | very serious^e^ | publication bias strongly suspected^c^ | 37 | 38 | - | MD **0.36 higher**  (3.46 lower to 4.17 higher | ⨁◯◯◯  Very low | IMPORTANT |
| **SOFA score^j^** | 7 | observational studies | not serious | not serious | not serious | serious^b^ | none | 110 | 162 | - | MD **1.71 lower**  (2.51 lower to 0.9 lower) | ⨁◯◯◯  Very low | IMPORTANT |
| **lactate level^i^** | 2 | randomized trials | serious^a^ | serious^g^ | not serious | very serious^d^ | publication bias strongly suspected^c^ | 60 | 60 | - | MD **0.51 mmol/L lower**  (1.15 lower to 0.12 higher) | ⨁◯◯◯  Very low | IMPORTANT |
| **lactate level^j^** | 7 | observational studies | not serious | very serious^h^ | not serious | serious^b^ | none | 178 | 213 | - | MD **0.48 mmol/L lower**  (0.83 lower to 0.14 lower) | ⨁◯◯◯  Very low | IMPORTANT |
| **IL-6 level^i^** | 1 | randomized trials | serious^a^ | not serious | not serious | serious^b^ | publication bias strongly suspected^c^ | 30 | 30 | - | MD **0.69 pg/ml fewer**  (1.21 fewer to 0.16 fewer) | ⨁◯◯◯  Very low | IMPORTANT |
| **IL-6 level^j^** | 4 | observational studies | not serious | not serious | not serious | serious^b^ | none | 74 | 101 | - | MD **0.77 pg/ml fewer**  (1.09 fewer to 0.46 fewer) | ⨁◯◯◯  Very low | IMPORTANT |

CI: confidence interval; WMD: weighted mean difference; OR: odds ratio; SOFA: Sequential Organ Failure Assessment; NE: norepinephrine; IL-6: interleukin-6

a. Most information is from studies at low or unclear risk of bias. Potential limitations are likely to lower confidence in the estimate of effect.

b. The optimal information size criterion is not met.

c. Published evidence is limited to a few small trials.

d. The optimal information size criterion is not met. The 95% CI overlaps no effect and fails to exclude important benefit.

e. The optimal information size criterion is not met. The 95% CI overlaps no effect and fails to exclude important harm.

f. The optimal information size criterion is met. But the 95% CI overlaps no effect and fails to exclude important benefit.

g. Substantial heterogeneity, of unequivocal importance

h. Considerable heterogeneity, of unequivocal importance

i. The evidence is from RCTs.

j. The evidence is from observational studies.

Appendix 9: Amendments to the information provided in the protocol

**Searches**

The statement on the protocol:

We will search the following databases from study inception to July 2022: PubMed, EMBASE, Web of Science, and the Cochrane Library; Chinese databases Sino-Med, China National Knowledge Infrastructure, Wanfang Database, and VIP Database for Chinese Technical Periodicals; and several major clinical research registration websites (ClinicalTrials.gov, Chinese Clinical Trial Registry, and International Clinical Trials Registry Platform). The search terms mainly include keywords and MeSH terms related to “sepsis” and “oXiris.”

Having obtained the initial results from our search strategy, titles and abstracts will be screened by two authors independently. Potentially eligible studies will then be assessed using the full text to ascertain relevance. Any discrepancies or conflicts will be resolved with consensus by the third author.

The actual process:

We complemented our previous search with a supplementary search of these 11 databases to collect all studies relevant to our research from July to November 2022. In effect, titles and abstracts were screened by three authors independently. Any discrepancies or conflicts will be resolved with consensus by the fourth author.

**Secondary outcomes**

The statement on the protocol:

Additional outcomes will include intensive care unit (ICU) and hospital mortality, length of ICU and hospital stay, and so on.

The actual process:

To better explain the occurrence of relevant clinical events, we decided to include new indicators, such as interleukin-6 (IL-6) level, norepinephrine (NE) dose, lactate level, and Sequential Organ Failure Assessment (SOFA) score.

**Strategy for data synthesis**

The statement on the protocol:

We will investigate clinical heterogeneity by subgroup analyses on types of control filters and so on.

The actual process:

In our analysis, we found little heterogeneity among most indicators. In the heterogeneity test of the meta-analysis of seven outcomes, I^2^ was even 0%, suggesting homogeneity. Therefore, we decided not to conduct subgroup analyses based on the control filter type. However, when rating the quality of the evidence with the grading of recommendations assessment, development, and evaluation approach, the quality of the evidence differed between observational studies and randomized controlled trials (RCTs). Therefore, we conducted a subgroup analysis to explore whether the results of the studies varied.

Appendix 10: The baseline similarity


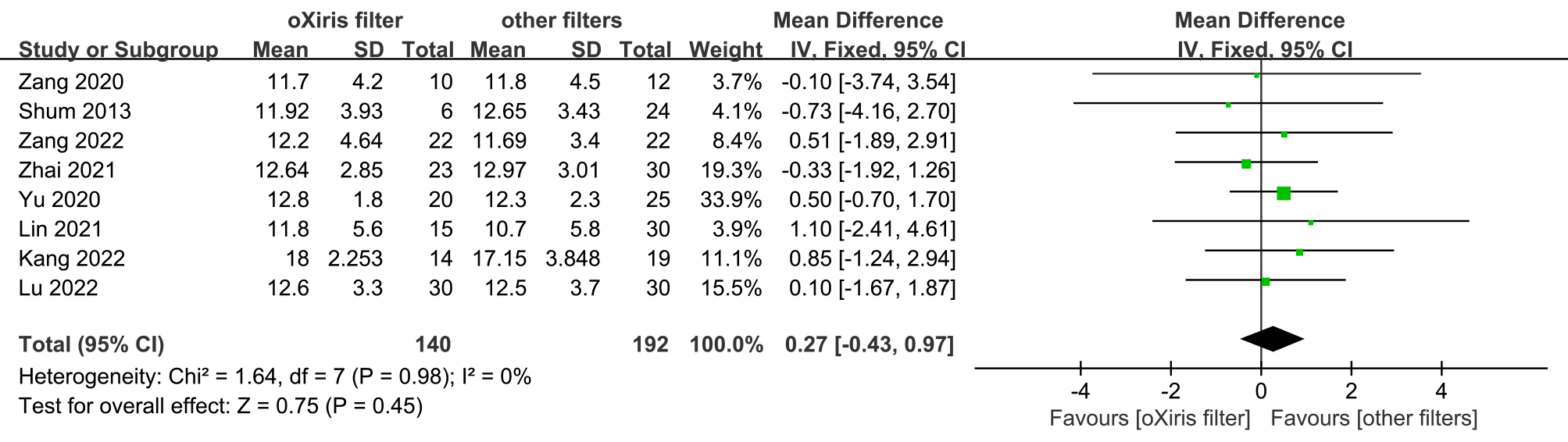


Fig. S27 SOFA score at baseline


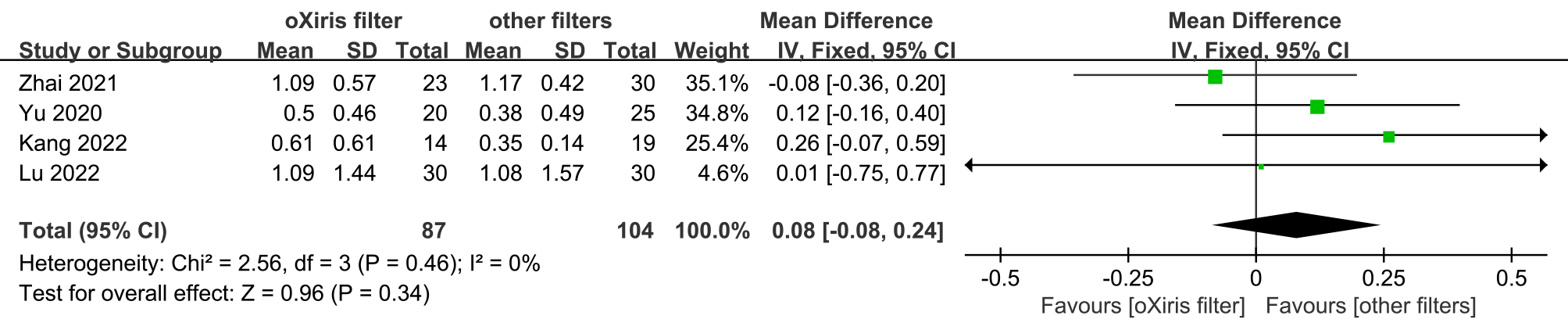


Fig. S28 NE dose at baseline


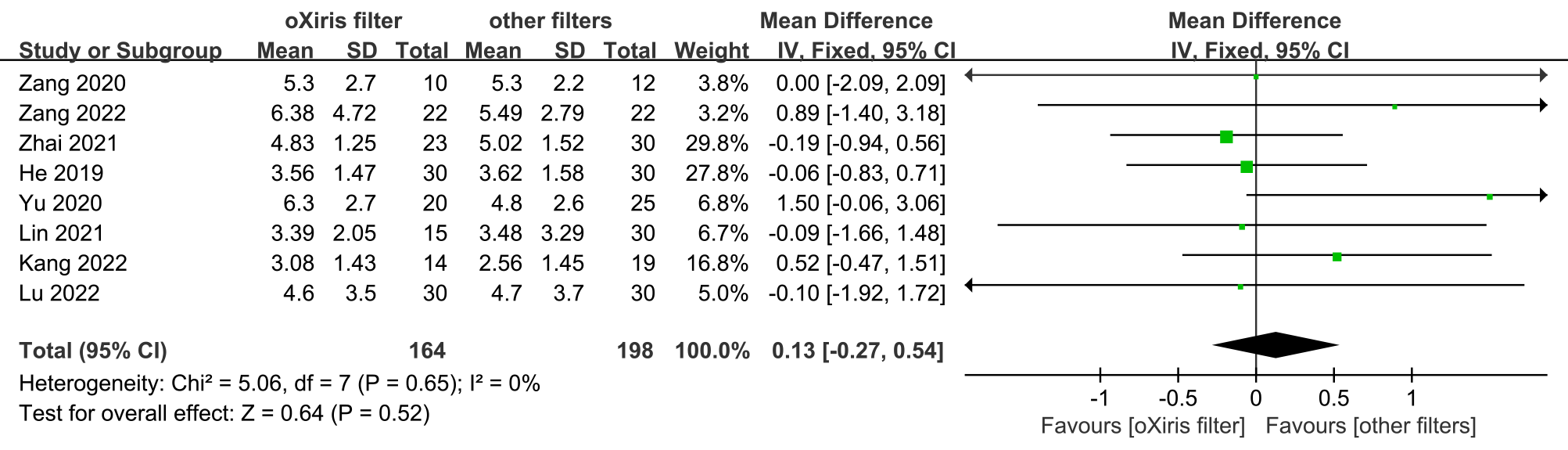


Fig. S29 Lactate level at baseline


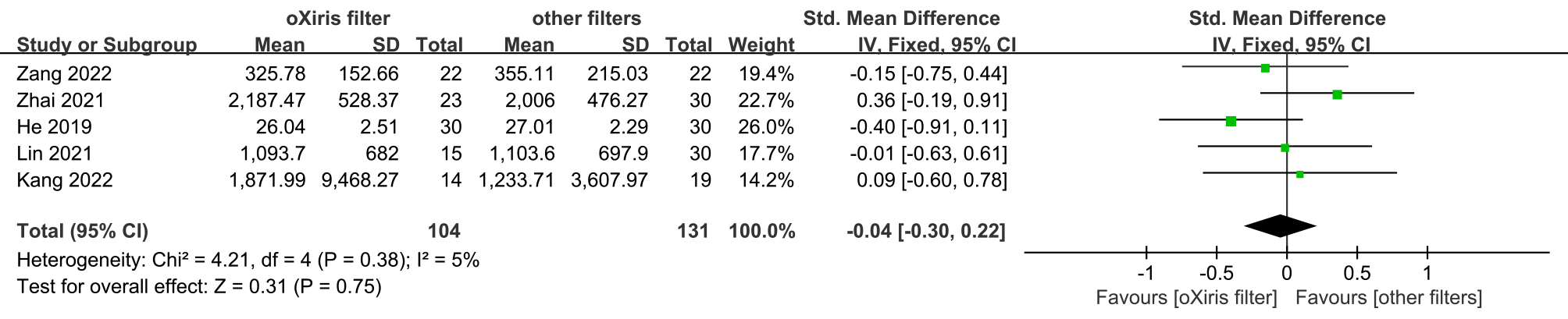


Fig. S30 IL-6 level at baseline


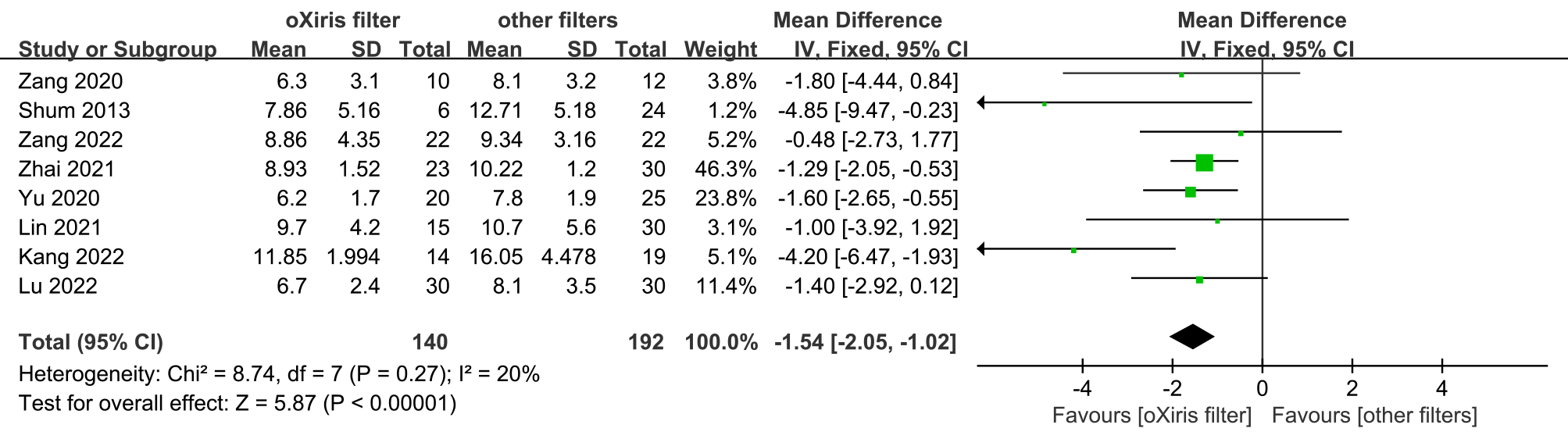


Fig. S31 SOFA score after treatment (excluding Broman’s study)


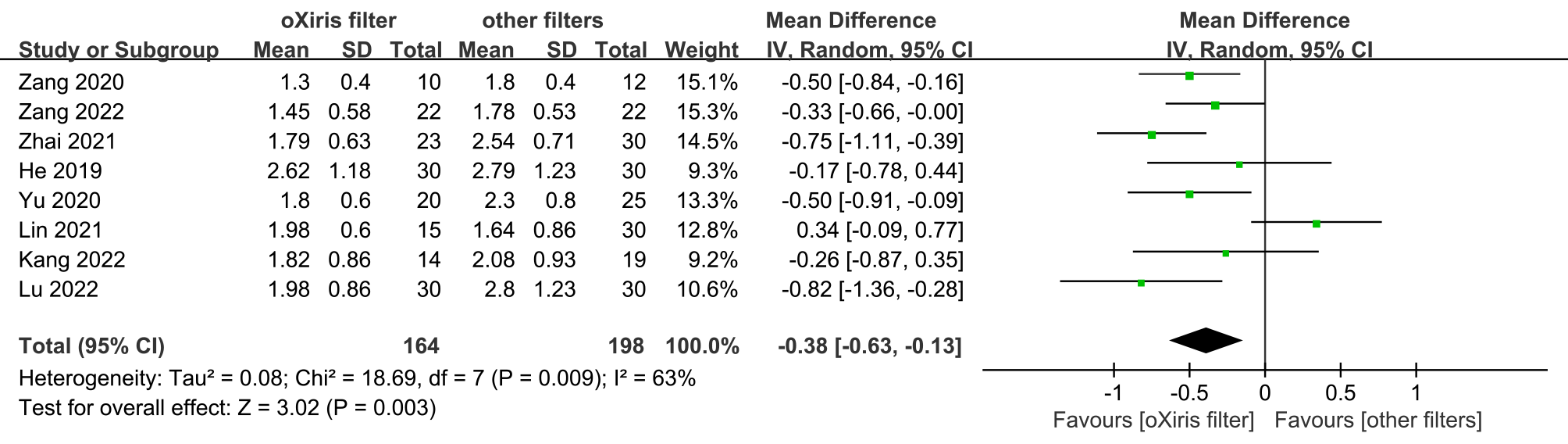


Fig. S32 lactate level after treatment (excluding Xie’s study)

**References**

1. Kobayashi S, Ohtake T. The Characteristics of Dialysis Membranes: Benefits of the AN69 Membrane in Hemodialysis Patients. J Clin Med. 2023;12(3). doi:10.3390/jcm12031123.

2. Broman ME, Hansson F, Vincent JL, Bodelsson M. Endotoxin and cytokine reducing properties of the oXiris membrane in patients with septic shock: A randomized crossover double-blind study. PLoS One. 2019;14(8):e0220444. doi:10.1371/journal.pone.0220444.

3. Zhang Q, Wang L, Liu B, Cai X, Qi Z, Sun Z. Application of FX60 and FX80 dialyzer on complications in maintenance hemodialysis patients. China Journal of Modern Medicine. 2014;24(32):73-6. doi:10.3969/j.issn.1005-8982.2014.32.016. (in Chinese).

4. Kameshwar K, Damasiewicz MJ, Polkinghorne KR, Kerr PG. A pilot study comparing the efficiency of a novel asymmetric cellulose triacetate (ATA) dialyser membrane (Solacea-190H) to a standard high flux polysulfone dialyser membrane (FX-80) in the setting of extended hours haemodialysis. Nephrology (Carlton). 2022;27(6):494-500. doi:10.1111/nep.14030.

5. Zang S, Chen J, Zhang Y, Chen Q, Xu L. Application effects of AN69-ST and AN69-oXiris filters in patients with sepsis. Guangxi Medical Journal. 2020;42(24):3183-6+96. (in Chinese).

6. Shum HP, Chan KC, Kwan MC, Yan WW. Application of endotoxin and cytokine adsorption haemofilter in septic acute kidney injury due to Gram-negative bacterial infection. Hong Kong Med J. 2013;19(6):491-7. doi:10.12809/hkmj133910.

7. Zang S, Chen Q, Zhang Y, Xu L, Chen J. Comparison of the Clinical Effectiveness of AN69-oXiris versus AN69-ST Filter in Septic Patients: A Single-Centre Study. Blood Purif. 2022;51(7):617-29. doi:10.1159/000519166.

8. Guan M, Wang H, Tang X, Zhao Y, Wang F, Zhang L, et al. Continuous Renal Replacement Therapy With Adsorbing Filter oXiris in Acute Kidney Injury With Septic Shock: A Retrospective Observational Study. Front Med (Lausanne). 2022;9:789623. doi:10.3389/fmed.2022.789623.

9. Xie J, Xiao W, Lin J. Effect of oXiris-CVVH on the Clinical Outcomes of Patients with Septic Shock: An Inverse Probability of Treatment-Weighted Analysis. Blood Purif. 2022:1-18. doi:10.1159/000524088.

10. Zhai Y, Pan J, Zhang C. The application value of oXiris-endotoxin adsorption in sepsis. Am J Transl Res. 2021;13(4):3839-44.

11. Le Y, Wang W. Effect of different filters on the prognosis of patients with septic shock: a single-center study. Chinese Journal of Emergency Medicine. 2022;31(05):683-6. (in Chinese).

12. Yu Z, Ran X, Li S. Application of endotoxin adsorption hemofilter continuous renal replacement therapy in septic shock patients with acute kidney injury. Chinese Journal of Blood Purification. 2020;19(12):798-802. (in Chinese).

13. Lin X. Efficacy of continuous renal replacement therapy with the oXiris filter in the treatment of patients with sepsis combined with acute kidney injury [Master's thesis]: ChongQing Medical University. (in Chinese); 2021.

14. Kang S. The effectiveness of oXiris-endotoxin adsorption treatment in patients with septic shock [Master's thesis]: Xinjiang Medical University. (in Chinese); 2022.

15. Feng J, Zhang S, Ai T, Wang L, Gao Y, Li W, et al. Effect of CRRT with oXiris filter on hemodynamic instability in surgical septic shock with AKI: A pilot randomized controlled trial. Int J Artif Organs. 2022;45(10):801-8. doi:10.1177/03913988221107947.

16. He W. Evaluation of two hemofiltration membranes in the intervention and effect of inflammatory mediators in sepsis treatment by hemodialysis [Master's thesis]: China: Qinghai University. (in Chinese); 2019.

17. Lu T, Cai Y, Zhang L, Wang J. Clinical Effect of OXiris-Endotoxin Adsorption on Septic Shock Patients. Clinical Research. 2022;30(09):101-3. (in Chinese).
